# Arbuscular mycorrhizal fungi enhance active ingredients of medicinal plants: a quantitative analysis

**DOI:** 10.3389/fpls.2023.1276918

**Published:** 2023-10-20

**Authors:** Ming-Li Yuan, Meng-Han Zhang, Zhao-Yong Shi, Shuang Yang, Meng-Ge Zhang, Zhen Wang, Shan-Wei Wu, Jia-Kai Gao

**Affiliations:** ^1^ College of Agriculture, Henan University of Science and Technology, Luoyang, Henan, China; ^2^ School of Agriculture and Animal Husbandry Engineering, Zhoukou Vocational and Technical College, Henan, China; ^3^ Henan Engineering Research Center for Rural Human Settlement, Luoyang, Henan, China; ^4^ Luoyang Key Laboratory of Symbiotic Microorganism and Green Development, Luoyang, Henan, China

**Keywords:** arbuscular mycorrhizal fungi, medicinal active ingredients, medicinal plant, secondary metabolites, physiological variables

## Abstract

Medicinal plants are invaluable resources for mankind and play a crucial role in combating diseases. Arbuscular mycorrhizal fungi (AMF) are widely recognized for enhancing the production of medicinal active ingredients in medicinal plants. However, there is still a lack of comprehensive understanding regarding the quantitative effects of AMF on the accumulation of medicinal active ingredients. Here we conducted a comprehensive global analysis using 233 paired observations to investigate the impact of AMF inoculation on the accumulation of medicinal active ingredients. This study revealed that AMF inoculation significantly increased the contents of medicinal active ingredients by 27%, with a particularly notable enhancement observed in flavonoids (68%) and terpenoids (53%). Furthermore, the response of medicinal active ingredients in belowground organs (32%) to AMF was more pronounced than that in aboveground organs (18%). Notably, the AMF genus *Rhizophagus* exhibited the strongest effect in improving the contents of medicinal active ingredients, resulting in an increase of over 50% in both aboveground and belowground organs. Additionally, the promotion of medicinal active ingredients by AMF was attributed to improvements in physiological factors, such as chlorophyll, stomatal conductance and net photosynthetic rate. Collectively, this research substantially advanced our comprehension of the pivotal role of AMF in improving the medicinal active ingredients of plants and provided valuable insights into the potential mechanisms driving these enhancements.

## Introduction

The knowledge surrounding medicinal plants represents the culmination of wisdom accumulated over thousands of years (Petrovska, 2012; Zuo et al., 2020). It is recorded that humans began utilizing medicinal plants for treating diseases as early as 2,500 years ago (Wiart, 2006). The efficacy of medicinal plants has gained global recognition (Shen et al., 2022; Wang et al., 2022b). World Health Organization (WHO), (2019) reported that more than 80% of the populations in the world regularly relies on medicinal plants to combat various. It is also well known that the extraction of artemisinin from medicinal plants by Tu Youyou earned her the prestigious Nobel Prize in 2014. Artemisinin had successfully treated 22.395 million individuals by 2018, as reported by World Health Organization (WHO), (2022). Furthermore, extracts from medicinal plants such as quercetin, luteolin and acacetin have shown significant potential in enhancing the immunity of COVID-19 patients and reducing the likelihood of entering the critical stage (Luo et al., 2020; Ren et al., 2020; Runfeng et al., 2020; Khadka et al., 2021). However, in recent years, the quality of medicinal plant has experienced a sharp decline alongside the increase in cultivation yield. This decline manifests in decreased contents of medicinal active ingredients and weakened physiological activities (Zeng et al., 2013a; Wang et al., 2018). In response, various scholars have undertaken studies involving different approaches such as the use of fertilizers (Alaoui Jamali et al., 2014) and breeding techniques (Wang et al., 2020) to improve the quality of medicinal plants. Among these approaches, the use of arbuscular mycorrhizal fungi (AMF) as a green fertilizer has garnered significant attention for its potential in enhancing the quality of medicinal plants.

AMF as a ubiquitous soil microorganism plays irreplaceable roles in the management of plant quality (Davison et al., 2015; van der Heijden et al., 2015; Noceto et al., 2021). AMF enhances plant quality traits by regulating plant metabolic activity and chemical compounds, including soluble sugars, polysaccharides, flavonoids, and alkaloids (Grimoldi et al., 2006; Verlinden et al., 2018; Zhang et al., 2020a; Medina et al., 2023). Moreover, compounds such as flavonoids and alkaloids can also serve criteria for evaluating the quality of medicinal plants (Zhang et al., 2020a). It has been reported that more than 90% of medicinal plant species establish symbiotic associations with AMF (Zhang et al., 2022). Recent studies have shown that AMF can promote medicinal plant seed germination (Zhang et al., 2020b), growth (Luo et al., 2021) and secondary metabolic activity (Zeng et al., 2013b), and enhance phenols (Duc et al., 2021), flavonoids (Zhang et al., 2020a), terpenes (Xie et al., 2018a), quinones (Yang et al., 2017) and other medicinal active ingredients (Zeng et al., 2013b). AMF are also well known to help plants tolerate various environmental stressors, such as drought, salinity, or heavy metal toxicity (Porcel et al., 2015; Zhao et al., 2022). Stress mitigation indirectly supports active ingredient production by maintaining plant health. The research on improving the quality of medicinal plants by AMF inoculation has become one of the hotspots (Jeffries et al., 2003; Gosling et al., 2006; van der Heijden et al., 2015; Amani Machiani et al., 2022; Abdullahi et al., 2023).

Currently, a large number of studies on AMF and medicinal plants have been conducted to explore the effects of mycorrhizal symbiosis on medicinal active ingredients (Zeng et al., 2013b; Ostadi et al., 2020; Duc et al., 2021; Zhao et al., 2022). Most studies found that AMF inoculation increased medicinal active ingredient contents in plants (Srivastava et al., 2016; Chen et al., 2017; Xie et al., 2018a), and a few researches reported AMF had negative or no effects on medicinal active ingredients (Vieira et al., 2021). The effects of AMF inoculation on medicinal active ingredients differed from organs (aboveground and belowground organs) and medicinal components (Zubek et al., 2012; Lima et al., 2017; Xie et al., 2018b). While, the quantitative effect of AMF inoculation on medicinal active ingredients remains unclear. Filling this critical knowledge gap will help to provide a theoretical reference for enhancing the medicinal active ingredients by AMF inoculation and refine the theory of mycorrhizal symbiosis.

AMF colonization is widely perceived as one of the key ways to impact the medicinal active ingredients in plants (Zeng et al., 2013a; Zeng et al., 2013b). Previous comprehensive studies have shown the potential of AMF in enhancing medicinal active ingredients (Mandal et al., 2013; Zeng et al., 2013a; Zeng et al., 2013b). However, the responses of medicinal active ingredients in plant organs to AMF were disparate (Xie et al., 2018b; Yu et al., 2019; Merlin et al., 2020), which may due to the differences of physiological activities in different plant organs when inoculated with AMF (Chen et al., 2017; Merlin et al., 2020). Therefore, it is necessary to extensively research the effects of medicinal active ingredients in plant organs interacting with AMF, to obtain the general response model from the specific response of medicinal active ingredients in plant organs to AMF inoculation.

Previous studies have shown that AMF is universal in improving the quality of medicinal active ingredients (Vo et al., 2019; Zhang et al., 2020a; Wu et al., 2021), such as enhancing the synthesis and accumulation of terpenes and flavonoids in medicinal plants (Hristozkova et al., 2016; Wei et al., 2020; Zhang et al., 2020a). While the efficacy of AMF promoting medicinal active ingredients was diverse (Zubek et al., 2012; Hristozkova et al., 2016; Wu et al., 2021) and there is no consensus on which medicinal active ingredients are most affected by AMF or which species of AMF had the best effect on medicinal active ingredients (Hristozkova et al., 2016; Chen et al., 2017; Lima et al., 2017). The selection of the optimal AMF to enhance medicinal active ingredients has been a prominent and widely discussed topic (Lima et al., 2017; Khalediyan et al., 2021; Wu et al., 2021). Medicinal active ingredient has different responses to different AMF species (Andrade et al., 2012; Yu et al., 2019). Single or multi-AMF inoculation also has different effects on medicinal active ingredients (Zubek et al., 2012; Duc et al., 2021). Considering the inconsistent results, it is vital to synthesize the available data to reveal clear and generalizable patterns of the response of medicinal active ingredients to single or multi-AMF inoculation, and to further identify the AMF species with the most significant effects on medicinal active ingredients.

In this study, we conducted a quantitative analysis at the first time to assess the effect of AMF on medicinal active ingredients. We hypothesized that (a) the effect of AMF on medicinal active ingredients linked with plant organ and compounds of medicinal active ingredients, (b) single or multi-AMF inoculation had different effects on medicinal active ingredients. The study aims to provide a reference to increase medicinal active ingredients by AMF inoculation and theoretical foundation for the development of the pharmaceutical industry.

## Materials and methods

### Search strategy and eligibility criteria

This meta-analysis followed the PRISMA protocol (Moher et al., 2015). Peer-reviewed publications researching on AMF associated with medicinal plants and measured indices of medicinal active ingredients were searched using Web of Science database until December 2021 with no restriction on publication year. The search terms were ‘(arbuscular mycorrhiza) AND (active ingredients OR secondary metabolic product)’. Papers included in the analysis had to meet the following criteria: (a) the papers had to be original research and reviews were excluded; (b) experiments involved AMF treatment and a corresponding control; (c) the object of study was a medicinal plant; (d) at least one medicinal active ingredient was included in the study; (e) the number of replicates for AMF treatments and control groups can be obtained; (f) means or standard deviations (SD) or standard errors (SE) or 95% confidence interval of the AMF treatment and control groups can be obtained.

### Data extraction

**Figure 1:**
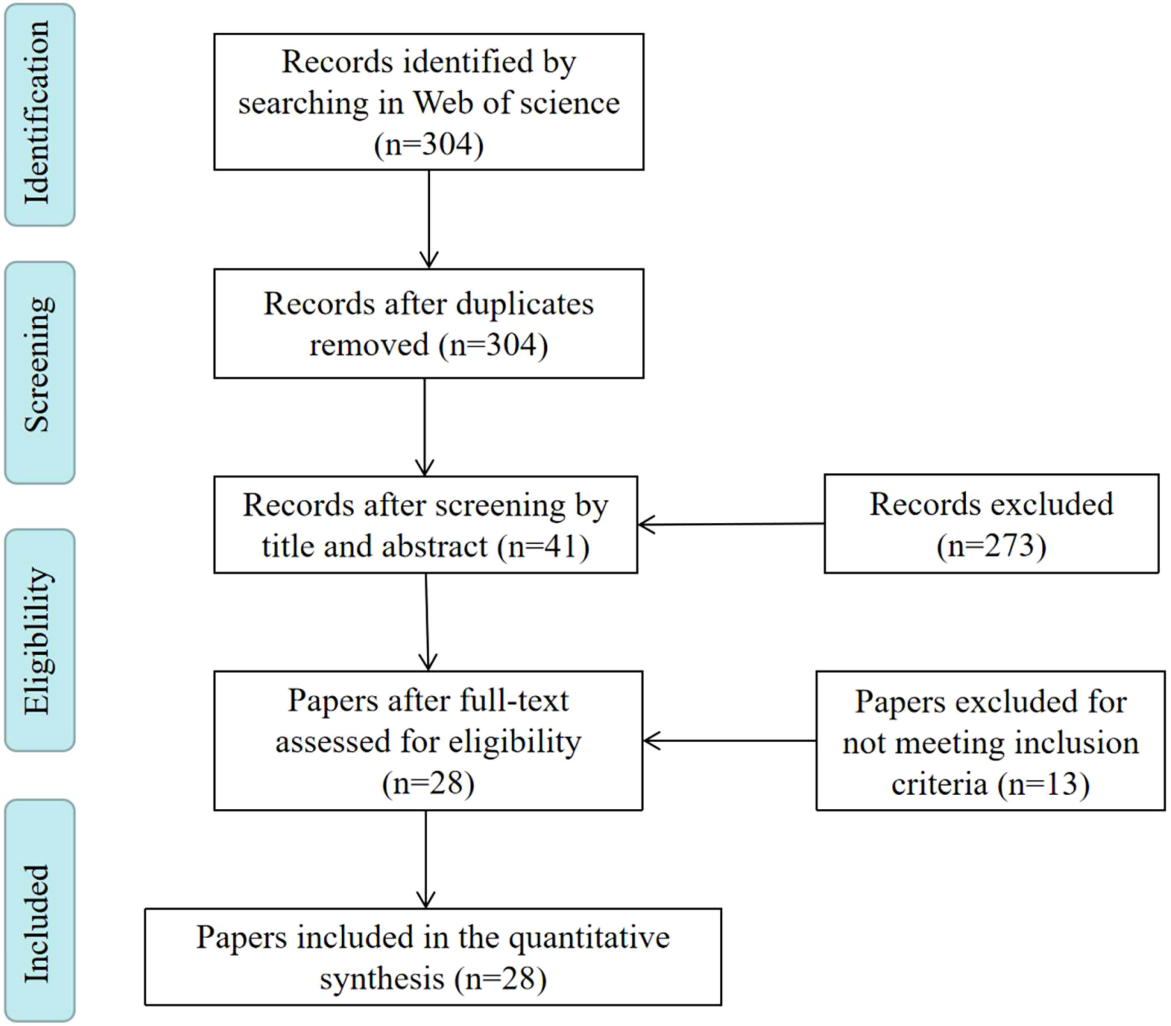
Flowchart of literature selection process.

For the quantitative analyses, each included publication was evaluated by two reviewers (Menghan Zhang and Mingli Yuan) independently and for accuracy the searching results were cross-checked. In case of any discrepancies, a third reviewer (Zhaoyong Shi) was involved. Based on the search strategy, 28 papers were included in this systematic analysis (**Figure 1**, **Supplementary Data 1**).

According to Han et al. (2020) and Luo et al. (2006), we obtained data on medicinal active ingredient information for each study and physiological variables if reported. All original data were obtained from main text, tables, figures and **Supplementary Files** of literature. When the data were presented in figures, we extracted numerical data using GetData Graph Digitizer 2.26. In total, our database included 233 observations on medicinal active ingredients (detailed information were shown in **Table S1**).

For each observation, the components of medicinal active ingredients were classified into phenols, flavonoids, alkaloid, terpenes, organic acids, quinones and others with aboveground or belowground organs and single- or multi-AMF recorded. About single AMF species classification, we referred to the website (http://www.speciesfungorum.org/). The effect of AMF inoculation on medicinal active ingredient were analyzed from order, family and genus level. Due to data limitations, we did not study the impact of AMF at the species level and focusing on the genus level allows us to address a broad range of AMF species and their potential effects on multiple medicinal plant species. In order to investigate the physiological mechanism of AMF effecting medicinal active ingredient, we also recorded seven physiological variables including total carotenoids (TC), chlorophyl A (Chl-A), chlorophyl B(Chl-B), total carbohydrates (CHO), stomatal conductivity (Gs), net photosynthetic rate (Pn) and water utilization ratio (WUE) (detailed information were shown in **Table S2**).

### Data analysis

The effect size of medicinal active ingredients to AMF was evaluated by the natural log response ratio (lnRR) (Hedges et al., 1999; Luo et al., 2006). The response ration was calculated as:


(1) $$\text{ln}RR=\text{ln}(X_{T}/X_{C})\;=\text{ln}(X_{T})\;–\text{ln}(X_{C}),$$

Where *X*_T_ and *X*_C_ represent the medicinal active ingredients in AMF treatment and the control treatment, respectively.

The variance (*v*) of every ln*RR* was calculated as follows:


(2) $$vi=\frac{S_{T}^{\;2}}{n_{T}X_{T}^{\;2}}+\frac{S_{C}^{\;2}}{n_{C}X_{C}^{\;2}},$$

Where *S*_T_ and *S*_C_ represent *SD* of AMF treatment and control treatment, respectively, *n*_T_ and *n*_C_ are the sample sizes.

The conversion equation for *SD* and *SE* is as follows:


(3) $$SD=SE\sqrt{n},$$

Where *n* is the sample size. In several studies with no *SD* or *SE* and only 95% confidence intervals, we identified 1/10 of the mean as *SD* (Han et al., 2020).

This study employed the Q test and *I*² statistic to assess heterogeneity among the included literature. In the Q test, a larger Q value and a smaller *P* value indicate significant heterogeneity when *P*<0.01. The *I*² statistic quantifies the degree of heterogeneity, with values exceeding 50% considered significant heterogeneity (Higgins et al., 2003). When *I*²<50%, a fixed-effects model is employed; when *I*²>50%, it is advisable to use a random-effects model. Based on heterogeneity analysis (**Tables S3**, **S4**), a random-effects model was employed in present study (Viechtbauer, 2010; Zhang et al., 2019).

The weighted response ratio and 95% confidence interval were calculated using “metafor” package in *R* 3.4.1 (http://R-project.org/). If the value of the confidence interval (95%) does not coincide with 0, the effect of AMF inoculation was considered significant (Han et al., 2020).

To explore the relationship between medicinal active ingredients effect and total physiological factors (TC, ChlA&B, Gs, CHO, Pn and WUE), linear regression analysis was performed by SPSS (Version 23.0, Chicago, IL, USA).

## Results

### The overall effects of AMF on medicinal active ingredients

**Figure 2:**
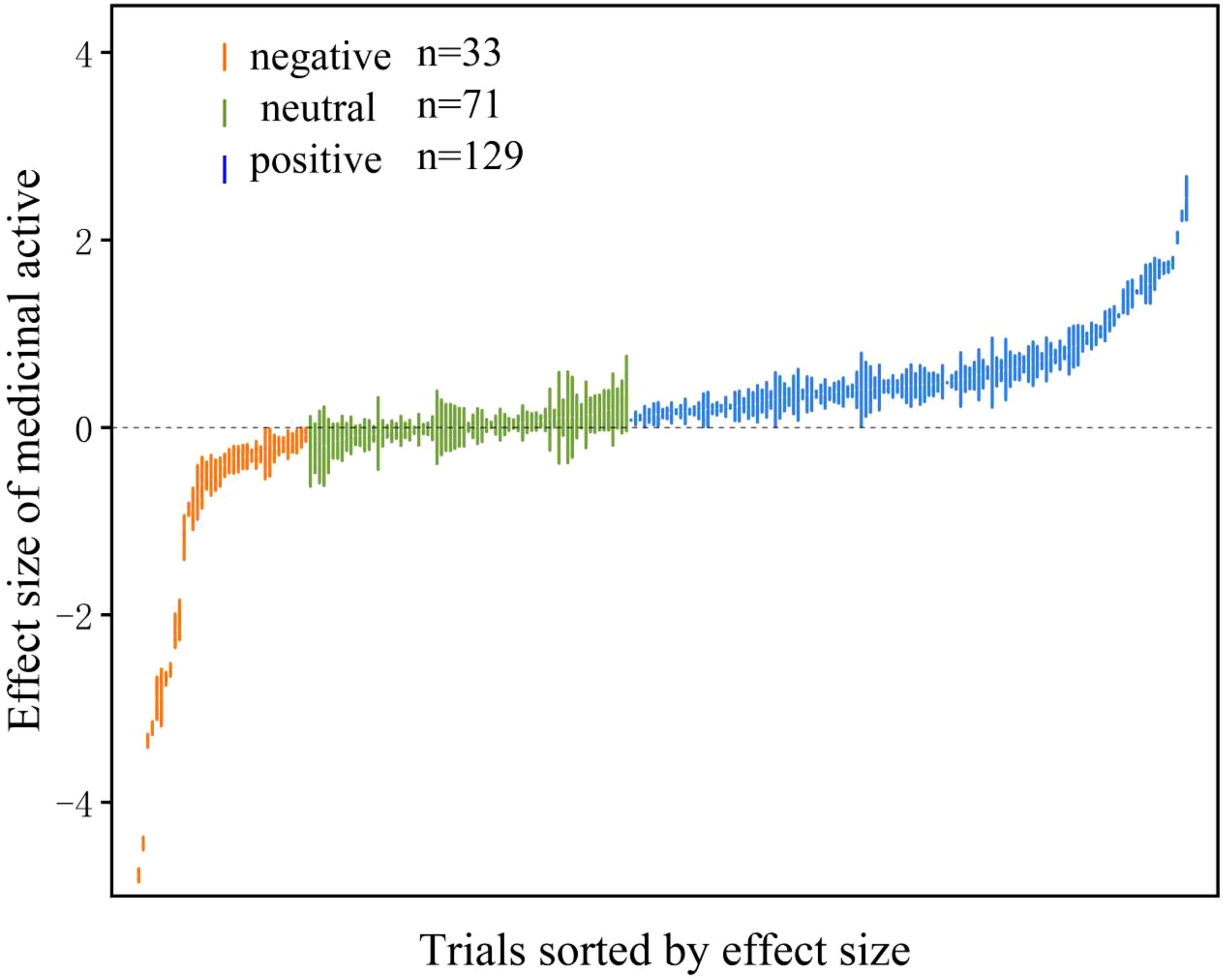
Effect of arbuscular mycorrhizal fungi (AMF) on medicinal active ingredient. The “*n*” represents trial numbers. The green, vermilion and blue data points represent the positive, neutral and negative effect, respectively.

**Figure 3:**
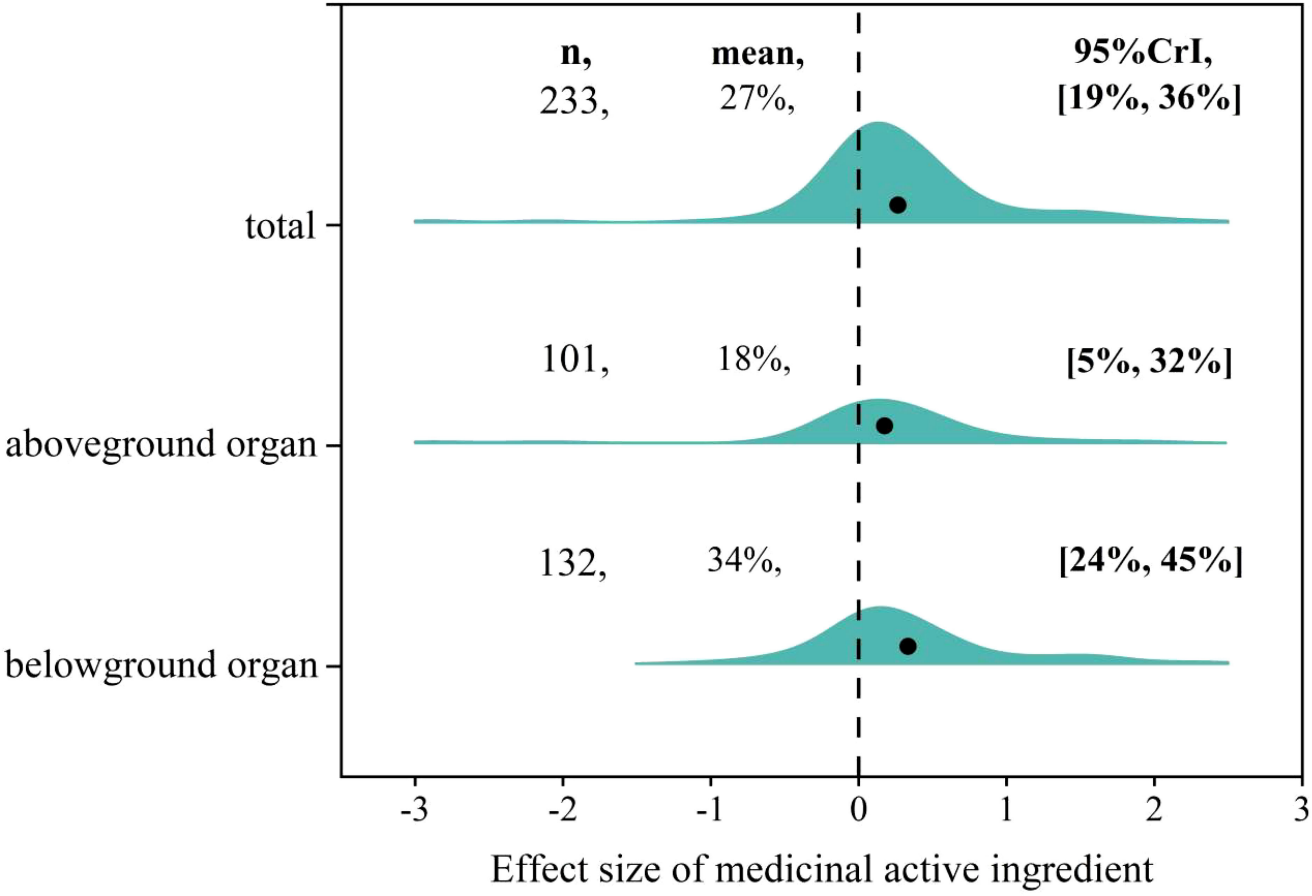
Overall effect of AMF on medicinal active ingredients including Total, in Aboveground and Belowground organs.

The effect size of AMF on medicinal active ingredients ranged from -2.89 to 2.44 with positive, negative and neutral effect size accounting for 55.4%, 14.2%, 30.4%, respectively (**Figure 2**). The overall mean effect size of AMF inoculation on medicinal active ingredients was 27% (CI [19-36%]) (**Figure 3**). When the plant organs were classified into aboveground and belowground organs, the result showed that the medicinal active ingredients both in aboveground and belowground organs were increased by AMF inoculation. The effect size of AM inoculation on belowground organs (18%, CI [5-32%]) was higher, but not significantly, than that on aboveground organs (34%, CI [24-45%]) (**Figure 3**).

### The overall effects of AMF on medicinal plants

In our current study, there are 20 different medicinal plant species across the 28 articles analyzed. From the list of the impact of AMF on these 20 medicinal plant species (**Table 1**), the findings revealed that AMF inoculation exerted a beneficial effect on the majority of these medicinal species. However, it should be noted that AMF inoculation appeared to have limited or ineffectual effects on a select few medical species, such as *Acmella oleacea* and *Eclipta prostrata*.

**Table 1:** List of beneficial or ineffective impact of AMF inoculation on medical plants.

Plant species	Medicinal active ingredients	Effect	Reference
*Acmella oleacea*	alkaloids	Ineffective	Vieira et al., 2021
*Artemisia annua*	terpenoids	Beneficial	Mandal et al., 2014
*Bituminaria bituminosa*	others	Ineffective	Pistelli et al., 2017
*Calendula officinalis*	phenols; flavonoids	Beneficial	Hristozkova et al., 2016
*Catharanthus roseus*	alkaloids; others	Ineffective	Andrade et al., 2013
*Commiphora leptophloeos*	phenols; flavonoids	Beneficial	Lima et al., 2017
*Dianthus superbus*	terpenoids	Beneficial	Yang et al., 2017
*Eclipta prostrata*	Phenols; Others	Beneficial/Ineffective	Duc et al., 2021; Vo et al., 2019
*Glycyrrhiza glabra*	terpenoids	Beneficial	Orujei et al., 2013
*Glycyrrhiza uralensis*	terpenoids	Beneficial	Chen et al., 2017; Xie et al., 2018; Xie et al., 2019; Yu et al., 2019
*Hypericum perforatum*	flavonoids; phenols	Beneficial	Lazzara et al., 2017; Zubek et al., 2012
*Myracrodruon urundeuva*	phenols	Beneficial	Barbosa da Silva et al., 2018
*Ocimum basilicum*	flavonoids; phenols	Beneficial	Hristozkova et al., 2018; Srivastava et al., 2016
*Panax quinquefolius*	terpenoids	Beneficial	Ran et al., 2021
*Passiflora edulis*	flavonoids	Beneficial	de Oliveira et al., 2019
*Plantago lanceolata*	terpenoids	Beneficial	Fontana et al., 2009
*Plectranthus amboinicus*	terpenoids	Beneficial	Merlin et al., 2020
*Salvia miltiorrhiza*	phenols; organic acids; quinones	Beneficial	Chen et al., 2017; Yang et al., 2017; Wu et al., 2021
*Salvia officinalis*	terpenoids	Beneficial	Tarraf et al., 2017
*Stevia rebaudiana*	terpenoids	Beneficial	Mandal et al., 2013

### Responses of different medicinal active ingredients to AMF inoculation

**Figure 4:**
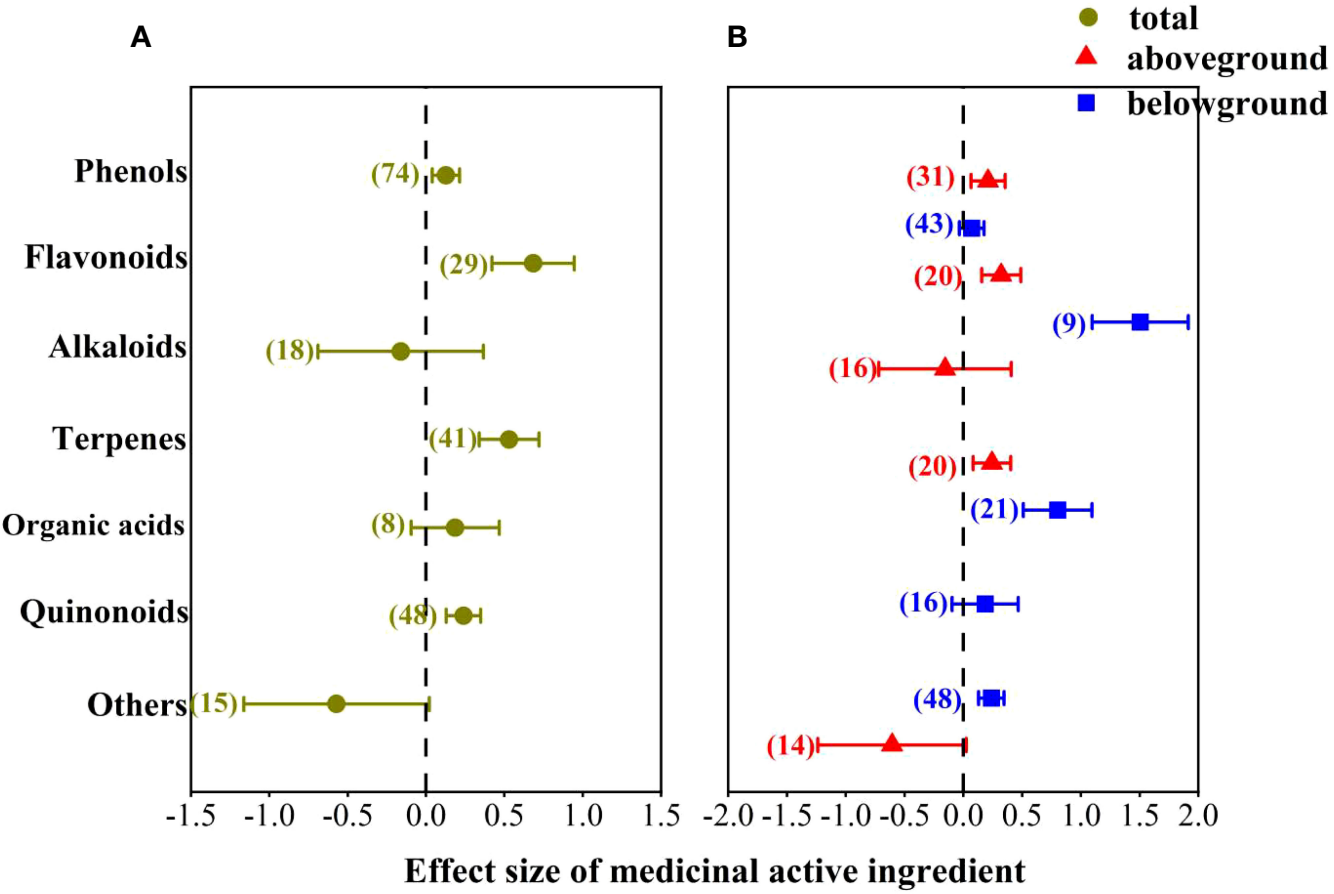
Effect of AMF on different compounds of medicinal active ingredients. **(A)** Total; **(B)** aboveground and underground organs.

We classified medicinal active ingredients into several kinds of compounds, such as phenols, flavonoids, alkaloid, terpenes, organic acids, quinones and others, to explore their responses to AMF inoculation. The results showed that AMF inoculation had prominent positive effects on flavonoids, terpenes, phenols and quinonoids (**Figure 4A**). The mean effect size on flavonoids was the highest (68%), followed by terpenoids (53%), and on phenols and quinones was 13% and 24%, respectively. The positive effect of AMF inoculation on organic acids was non-significant with an average effect size of 19%. When comes to alkaloids and others, the effect was negative, but not significant.

Furthermore, we further investigated the effects of AMF inoculation on different medicinal compounds in different organs (**Figure 4B**), the results were similar to the overall. It’s worth noting that the effect of AMF inoculation on flavonoids and terpenoids was more effective in belowground organs, with effect size 150% and 80%, respectively. The effect of AMF inoculation on phenols was more effective in aboveground organs with effect size 21%.

### The different function between single and multi-AMF inoculation

**Figure 5:**
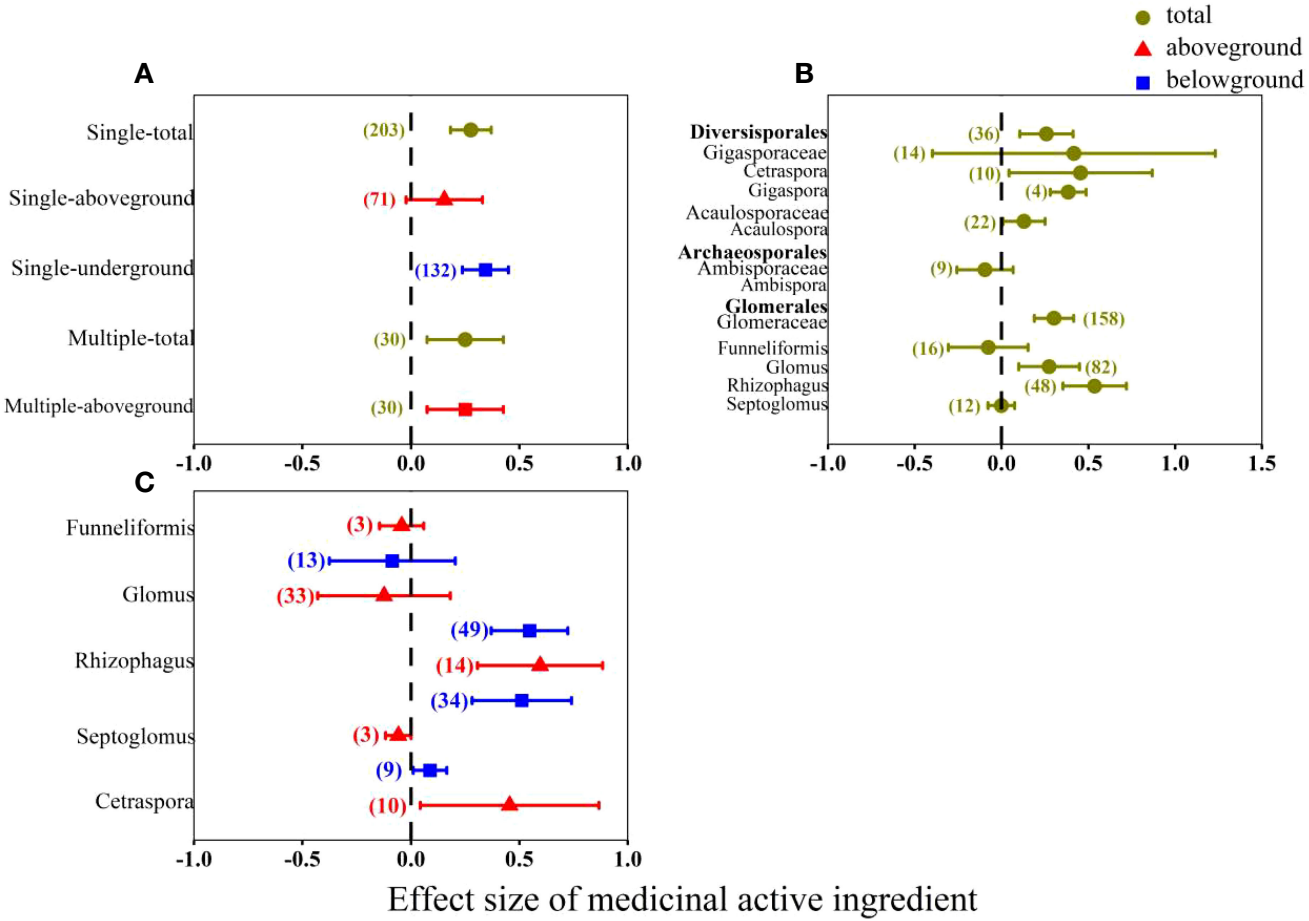
Effect of AMF on overall medicinal active ingredients. AMF classification: **(A)** signal or multi-AMF inoculation; **(B)** AMF inoculation on order, family and genus level; **(C)** AMF inoculation on genus level.

Result shows that the multi-AMF inoculation had significant effects on the medicinal active ingredients in aboveground organs with effect size of 25% (**Figure 5A**), while single AMF inoculation had insignificant positive effect. The single AMF inoculation significantly increased (34%) the contents of medicinal active ingredients in belowground organs. The study does not present the effect of multi-AMF inoculation on medicinal active ingredients in belowground organs, given the absence of relevant research on the subject. The effects of single AMF on the Order level were uneven (**Figure 5B**). Both Diversisporales and Glomerales had significant positive effects on medicinal active ingredients. And Archaeosporales had a non-significant negative effect on medicinal active ingredients. From AMF genus level, the *Rhizophagus* was the most effective genus that had the highest effect size on medicinal active ingredients both in aboveground (60%) and belowground organs (51%) compared with other genera (**Figure 5C**).

### Influence of AMF inoculation on physiological factors

**Figure 6:**
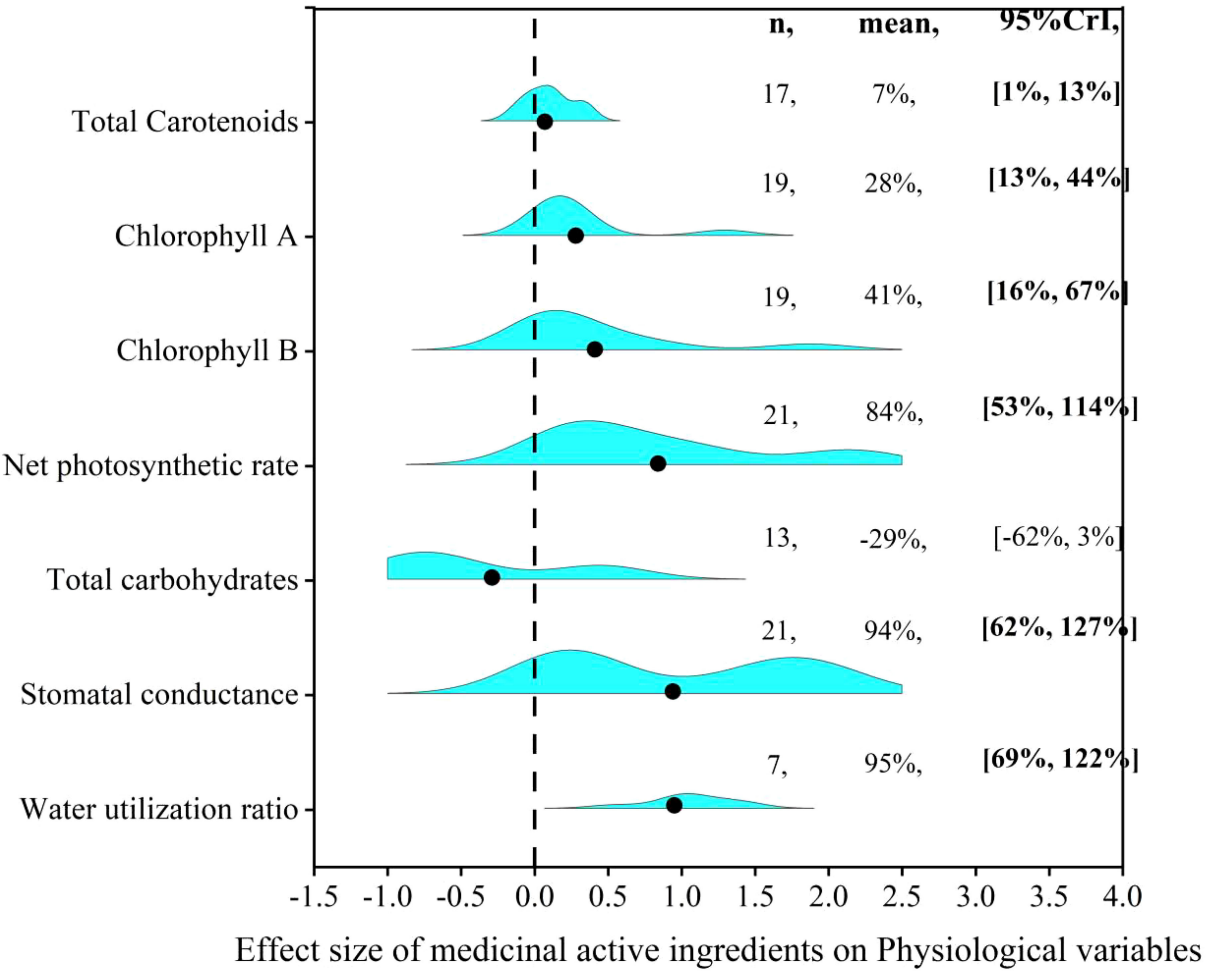
Effect of AMF on physiological factors.

**Figure 7:**
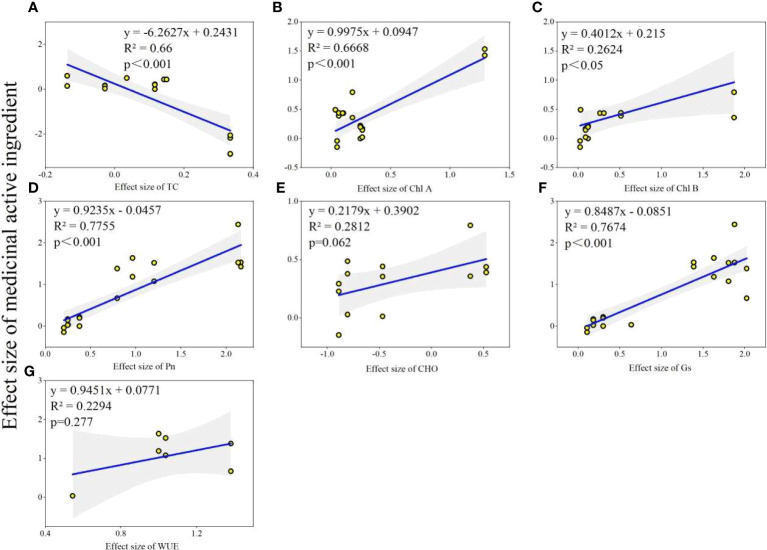
The relationship between effects sizes of medicinal ingredients and physiological factors. **(A)** TC: Total Carotenoids; **(B)** Chl A: Chlorophyll A; **(C)** Chl B: Chlorophyll B; **(D)** Pn: net photosynthetic rate; **(E)** CHO: total carbohydrates; **(F)** Gs: stomatal conductance; **(G)** WUE: water utilization ratio.

Physiological status of host plant was obviously improved by AMF inoculation (**Figure 6**). AMF inoculation significantly increased plant TC (6%), Chl A (28%), Chl B (41%), Pn (84%), Gs (94%) and WUE (96%). The results also showed that the AMF inoculation decreased CHO by 29%, but not significantly. Further, we explored the relationship between changes in plant physiological factors and changes in the content of medicinal active ingredients before and after AMF inoculation. We found that the effect size of medicinal active ingredients was positively correlated with Chl A (*R* ^2 = ^0.6668, *P*<0.001), Chl B (*R* ^2 = ^0.2424, *P*<0.005), Pn (*R* ^2 = ^0.7755, *P*<0.001), Gs (*R* ^2 = ^0.7674, *P*<0.001), but negatively correlated with TC (*R* ^2 = ^0.66, *P*<0.001) (**Figure 7**).

## Discussion

Based on a global scale quantitative synthesis with 233 pairs of data from 28 studies, we found that AMF inoculation have a significant positive effect on majority medicinal active ingredients, especially flavonoids in plant belowground organs. The enhancement of medicinal active ingredients by AMF inoculation may be related to the positive effect of AMF inoculation on plant physiological activity. By studying single or multi-AMF inoculation from different guilds, it was found that the medicinal active ingredients had the strongest response to *Rhizophagus*, one genus of AMF. Collectively, these findings provide a quantitative reference for the use of AMF inoculation to increase the content of medicinal active ingredients, which are valuable for the development of pharmaceutical industry.

Nowadays, human beings have a strong demand for the high quality medicinal active ingredients to better prevent and treat diseases. AMF has gained much attention because of its positive effect on medicinal active ingredients (Zeng et al., 2013b; Lima et al., 2017; Khalediyan et al., 2021). The quantitative analysis in this study confirmed that AMF inoculation could improve the quality of medicinal plants and enhanced the accumulation of medicinal active ingredients (De la Rosa-Mera et al., 2011; Mandal et al., 2013; Ran et al., 2021). In this study, we examined a total of 20 distinct medicinal plant species across the 28 articles under analysis. Our findings indicate that AMF inoculation predominantly exerts a favorable influence on the majority of these medicinal species, which will assist in guiding more targeted and effective AMF-based cultivation strategies for specific medicinal plant species. Furthermore, when investigating the interaction between AMF and plants, the concentration of AMF inoculation is also a crucial factor that should not be disregarded. Research indicates that the inoculation concentration of AMF can affect root system architecture (RSA). RSA traits such as total length, projected area, surface area, and volume tend to increase more significantly under higher AMF inoculation concentrations (Wu et al., 2012). The alteration in RSA induced by AMF may be associated with enhanced nutrient uptake (Padilla and Encina, 2005; Schroeder and Janos, 2005) and increased salt tolerance (Wu et al., 2011). Given the limited research on contrasting the effects of different concentrations of AMF inoculation on medicinal active ingredients, our study specifically centered on assessing whether AMF inoculation had an impact on them.

The findings that AMF enhanced the content of medicinal active ingredients in aboveground and belowground organs of plants were reported in previous researches (Srivastava et al., 2016; Xie et al., 2018a; Ran et al., 2021). Chen et al. proved that AMF inoculation significantly increased medicinal active ingredient contents in different belowground organs of *Glycyrrhiza uralensis* (Chen et al., 2017). Furthermore, Andrade et al. (2012) confirmed that AMF colonization significantly influenced the medicinal active ingredients in aboveground organs of *Catharanthus roseus*, compared to the medicinal plants with non-AMF colonization. Overall, consistent with our results in the present study, AMF inoculation has positive effects on medicinal active ingredient contents and the quality of medicinal plants. Scientific evidence has confirmed that the mycorrhizal network formed by AMF and plants increases the surface area for nutrient absorption, facilitating the uptake of essential nutrients such as phosphorus, nitrogen and various micronutrients (Smith and Read, 2008). Improved nutrient acquisition is fundamental for the biosynthesis of medicinal compounds.

The contribution of AMF to the increase of medicinal active ingredient contents varied depending on medicinal organs, medicinal compounds, and single or multi-AMF. AMF had the highest average effect size on medicinal active ingredient of the belowground organs, compared with that of aboveground organs. Previous studies have suggested that AMF have more potential in improving the productivity and ecological function of the belowground organs of plants (Hiiesalu et al., 2014; Yang et al., 2021). Yu et al. also found flavonoids in belowground organs of medicinal plants was significantly increased by *Rhizophagus* (Yu et al., 2019). Therefore, to further optimize the production of medicinal active ingredients in medicinal plants, it is advisable to inoculate with AMF when the belowground organs are intended for use in medicinal preparations, such as *Lonicera japonica, Coptis chinensis*, *Glycyrrhiza glabr*a, *Angelica sinensis* (Wei et al., 2016; Li et al., 2020; Sidhu et al., 2020; Tseng et al., 2022).

Single or multi-AMF inoculation had differed effects on medicinal active ingredients. Medicinal active ingredients with single AMF inoculation had the higher effect size compared to that with multi-AMF inoculation. Differences in the species and combinations of AMF were thought to be an impact factor on the medicinal active ingredient in previous studies (Zeng et al., 2013b; Lima et al., 2017; Vieira et al., 2021). Similar to our result that *Rhizophagus* had the best effect on increasing the concentration of medicinal active ingredients. It has been also proved to be a particularly excellent root colonizer and plant symbiotic compared to other species of AMF in the horticulture plants (Wang et al., 2022a). The genera may form particularly beneficial symbiotic relationships with medicinal plants, leading to increased production of secondary metabolites. Previous study also showed that *Rhizophagus* could improve quality of *Commiphora leptophloeos* by promoting the production of the secondary metabolites (Lima et al., 2017). Currently, little is known about the interactions between AMF and medicinal plants, but available data seem to support the idea that management of AMF rhizosphere communities may have important implications for the development of sustainable agriculture and more efficient production of medicinal plant materials (Gianinazzi et al., 2010).

The mechanism of AMF inducing changes in the concentration of medicinal active ingredients in different parts of medicinal plants can be multifaceted (Toussaint, 2007). Among them, one of the reasons is that the physiological and secondary metabolic ability of medicinal plants in the symbiotic state can be improved (Oliveira et al., 2013; Zeng et al., 2013b). Among the mentioned medicinal active ingredients in this study, flavonoids showed the strongest responses to AMF, followed by terpenoids. Similarly, previous publication reported that AMF symbiosis increased the accumulation of medicinal active ingredients, especially terpenoids and flavonoids (Xie et al., 2018b). In present study, the results showed that AMF inoculation had positive effects on most of physiological factors, such as TC, Chl, Pn, Gs and WUE. Consequently, the enhancement of medicinal active ingredients by AMF may be related to its promotion of physiological activities (Xie et al., 2018b; Yu et al., 2019; Ran et al., 2021).

Photosynthesis is one of the key critical physiological and metabolic activities in plants, and it is closely coupled with upstream and downstream physiological metabolic activities (Ferreira et al., 2004). TC and Chl are proxies of plant photosynthesis and reflect the physiological and metabolic activity of plants (Andrade et al., 2012). Abdel Latef & Chaoxing (2011) reported that the content of Chl A and Chl B were enhanced in *Lycopersicon esculentum* when inoculated with AMF. Ran et al. (2021) reported that the promotion of active ingredients in medicinal plants of *American ginseng* was closely related to Chl. It is well known that the photosynthesis of plants is affected by the conductance of Gs (Gago et al., 2016). The Gs of the plant further affects the Pn (Mathur et al., 2018). Pn is commonly used to characterize the intensity of photosynthetic physiological activity in plants (Xie et al., 2018a; Xie et al., 2018b). Xie et al. (2018b) showed that there was a certain relationship between the increase of photosynthetic intensity and the increase of medicinal active components of *Glycyrrhiza uralensis*. Compared with the control group, AMF inoculation increased photosynthetic physiological activity, but decreased the photosynthetic product CHO in our study. CHO is one of the most important products of plant physiological metabolism and one of the key exchanges of plant roots and AMF (Marschner and Dell, 1994). We speculate that symbiosis enhances the exchange of AMF and medicinal plant CHO, stimulates photosynthesis, and enhances physiological and metabolic activities of medicinal plants (Gupta et al., 2021). Moreover, the effect of medicinal plants on WUE was especially increased by AMF inoculation. The physiological and metabolic activities of plants are inseparable from the participation of water, and a higher WUE indicates a higher potential of physiological and metabolic activities (Suter et al., 2019). The studies indicate that an WUE in medicinal plants may serve as a mechanism through which mycorrhizas facilitate the accumulation of medicinal active ingredients. This is supported by the observation that an increased WUE in AMF-plants correlates with higher levels of medicinal active ingredients, regardless of the specific mycorrhizal species or plant species involved (Xie et al., 2018b).

Numerous studies have shown that the physiological changes, such as Chl, Pn and Gs in medicinal plants inoculated with AMF is the reason for the quality improvement of medicinal active ingredients (Xie et al., 2018a; Xie et al., 2018b; Suter et al., 2019; Ran et al., 2021). In this study, the positive relationship between the effects of AMF inoculation on medicinal active ingredients and physiological activities provided evidence that the improvement of physiological and metabolic activities contributes to the accumulation of medicinal active ingredients of plants. These effects are attributed to the complex interactions between AMF and the plant host. Chl play critical roles in photosynthesis and are essential for capturing and converting light energy into chemical energy. The improved Chl content can lead to increased photosynthetic efficiency and, subsequently, higher production of medicinal active compounds (Lichtenthaler, 1987; Blankenship, 2021). Gs is influenced by AMF through its impact on water and nutrient uptake. AMF can enhance plant’s water absorption capabilities by extending its root system through mycorrhizal hyphae (Zhao et al., 2022; Kuyper and Jansa, 2023). As a result, plants with AMF colonization tend to maintain more optimal stomatal conductance even under water-limiting conditions. AMF colonization often leads to an increased Pn, which is due to several factors, including improved nutrient availability (particularly P), enhanced water uptake, and the modulation of plant hormones by AMF (Evelin et al., 2009; Smith and Smith, 2011). The higher Pn provides more energy and carbon compounds for the biosynthesis of medicinal compounds.

In addition, the molecular-level mechanisms through which AMF regulate the synthesis of medicinal compounds are also receiving increasing attention. Research has demonstrated that secondary metabolites of plant root, serving as signal molecules, play a pivotal role in both establishing AMF symbiosis and triggering a defense response in plants, resulting in heightened secondary metabolite production (Vierheilig, 2004). Previous study found that flavonoids enhanced AMF invasion and infection rates, leading to increased secondary metabolite production in plants (Scervino et al., 2007). Strigolactone, categorized as a sesquiterpene, has been identified as a signaling molecule that fosters mycorrhizal symbiosis (Mitra et al., 2021). Besides, AMF colonization leads to changes in enzyme activities within host plants and has an impact on the expression of genes related to secondary metabolites (Shrivastava et al., 2015; Ran et al., 2022). For example, the transcription of genes responsible for isoprenoid biosynthesis was enhanced by AMF symbiosis and displayed a correlation with the quantitative levels of terpenoids (Mandal et al., 2015). In recent years, significant advancements have been made in researching the molecular-level mechanisms behind AMF-mediated enhancement of medicinal active ingredients (Mechri et al., 2015; Kapoor et al., 2017; Ran et al., 2022; Zhao et al., 2022). These developments offer fresh perspectives on enhancing the quality of medicinal plants through AMF utilization. Nevertheless, to fully unlock the potential of AMF in enhancing the quality of medicinal plants, further research in this area needs to be strengthened.

Nonetheless, there are limitations to the current study. First, our study analyzes the overall impact of AMF on medicinal plants, providing a broad perspective. It lacks specific AMF inoculation strategies tailored to individual plant species. Based on this foundation, we expect to establish more refined experiments to further screen AMF inoculation strategies that are suitable for local conditions. Additionally, it cannot be overlooked that soil quality also plays an irreplaceable role in the interaction between AMF and medical plants. Soil conditions, such as pH, temperature, available nitrogen and phosphorus etc., greatly impact the status of infection of AMF on medicinal plants (Zhao et al., 2012; Zeng et al., 2013b). AMF assist plants in enhancing mineral nutrition uptake and directly promote plant’s growth (Wei and Wang, 1991; Smith and Read, 2008; Zeng et al., 2013b; Soti et al., 2023). On the other hand, the AMF hyphae extend into the surrounding soil, potentially altering the soil structure, thereby contributing to the improvement of soil quality and fertility (Thirkell et al., 2017; Chen et al., 2018; Zhao et al., 2022). Consequently, these changes can have additional effects on medicinal plants. The interplay of soil quality, AMF, and medicinal plants involves both direct and indirect interactions, complicating the differentiation of their individual effects. In the current study, we focus solely on AMF as a variable to explore its influence on medicinal active ingredients. Therefore, the consideration of soil conditions will be encompassed in our future research endeavors.

## Conclusions

This study conducted a quantitative assessment of the effect of AMF inoculation on the active ingredients of medicinal plants on a global scale. The results showed a significant increase in the medicinal active ingredients of belowground organs following AMF inoculation, with flavonoids displaying the strongest positive response to AMF. Notably, the genus *Rhizophagus* had the highest effect size on medicinal active ingredients. Consequently, it is recommended to inoculate medicinal plants with the genus *Rhizophagus* to enhance the active ingredients, particularly when targeting active ingredients from belowground organs. Furthermore, when examining the relationships between the response of medicinal active ingredients and physiological variables, we found positive correlations between that active ingredients and Chl A, Chl B, Pn and Gs. In summary, the findings of this study provide valuable insights into the utilization of AMF to enhance the active ingredients of medicinal plants. However, further research is necessary to explore the impact of physiological activities and environmental factors on the medicinal active ingredients of AMF-inoculated medicinal plants, as well as the underlying mechanisms.

## Data availability statement

The original contributions presented in the study are included in the article/**Supplementary Material**, further inquiries can be directed to the corresponding author/s.

## Author contributions

M-LY: Data curation, Formal Analysis, Writing – original draft, Conceptualization, Methodology. M-HZ: Conceptualization, Writing – original draft. Z-YS: Conceptualization, Supervision, Writing – review & editing, Funding acquisition. SY: Methodology, Software, Writing – review & editing. M-GZ: Methodology, Software, Conceptualization, Writing – review & editing. ZW: Software, Writing – review & editing. S-WW: Data curation, Methodology, Writing – review & editing. J-KG: Software, Supervision, Writing – review & editing.
