# Supplementary material for: Arbuscular mycorrhizal fungi enhance active ingredients of medicinal plants: a quantitative analysis
Source: Front Plant Sci. 2023 Oct 20;14:1276918. doi: 10.3389/fpls.2023.1276918 (PMC10623335; doi:10.3389/fpls.2023.1276918)
Supplement: Supplementary file 1 [file DataSheet_1.pdf]

Supplementary Data 1:

Description of selected studies

Reference	Plant species	AMF inoculation	Plant organs	Compounds	Physiological factor
Andrade et al., 2013	*Catharanthus roseus*	S	A&B	alkaloids; others	/
Barbosa da Silva et al., 2018	*Myracrodruon urundeuva*	M	A	phenols	/
Chen et al., 2017a	*Glycyrrhiza uralensis*	S	B	terpenoids; flavonoids	/
Chen et al., 2017b	*Salvia miltiorrhiza*	S	A&B	phenols	/
Duc et al., 2021	*Eclipta prostrata*	M	A	phenols	/
Felix de Oliveira et al., 2019	*Passiflora edulis*	S	A	flavonoids	TC; Chl-A; Chl-B
Fontana et al., 2009	*Plantago lanceolata*	S	A	terpenoids	/
Hristozkova et al., 2016	*Calendula officinalis*	S	A	phenols; flavonoids	Pn; Gs; WUE
Hristozkova et al., 2018	*Ocimum basilicum*	S	A	flavonoids	/
Lazzara et al., 2017	*Hypericum perforatum*	M	A	phenols; flavonoids	/
Lima et al., 2017	*Commiphora leptophloeos*	S	A	phenols; flavonoids	CHO
Mandal et al., 2013	*Stevia rebaudiana*	S	A	terpenoids	Chl-A; Chl-B
Mandal et al., 2014	*Artemisia annua*	S	A	terpenoids	/
Merlin et al., 2020	*Plectranthus amboinicus*	S	A	terpenoids	/
Orujei et al., 2013	*Glycyrrhiza glabra*	S	B	terpenoids	/
Pistelli et al., 2017	*Bituminaria bituminosa*	S	A	others	TC
Ran et al., 2021	*Panax quinquefolius*	S	B	terpenoids	TC; Chl-A; Chl-B; Pn; Gs
Srivastava et al., 2016	*Ocimum basilicum*	S	B	phenols	/
Tarraf et al., 2017	*Salvia officinalis*	S	A	terpenoids	/
Vieira et al., 2021	*Acmella oleacea*	S	A	alkaloids	/
Vo et al., 2019	*Eclipta prostrata*	M	A	phenols; others	/

Wu et al., 2021	*Salvia miltiorrhiza*	S	B	phenols; organic acids; quinones	/
Xie et al., 2018	*Glycyrrhiza uralensis*	S	B	terpenoids	Pn;Gs;WUE
Xie et al., 2019	*Glycyrrhiza uralensis*	S	B	terpenoids; flavonoids	Pn; Gs
Yang at al., 2017b	*Dianthus superbus*	S	A	terpenoids	/
Yang et al., 2017a	*Salvia miltiorrhiza*	S	B	quinones	/
Yu et al., 2019	*Glycyrrhiza uralensis*	S	B	terpenoids	Chl-A; Chl-B; Pn; Gs
Zubek et al., 2012	*Hypericum perforatum*	S	A	flavonoids; phenols	/

Note: S: Single AMF inoculation; M: Multi-AMF inoculation; A: Aboveground organs; B: Belowground organs; AMF: arbuscular mycorrhizal fungi; Chl: chlorophyll; CHO: total carbohydrates; Pn: net photosynthetic rate; TC: total carotenoids; Gs: stomatal conductivity; WUE: water utilization ratio

List of selected papers

1. Andrade, S. A. L.; Malik, S.; Sawaya, A. C. H. F.; Bottcher, A.; Mazzafera, P. Association with arbuscular mycorrhizal fungi influences alkaloid synthesis and accumulation in Catharanthus roseus and Nicotiana tabacum plants. Acta Physiol Plant. 2013, 35(3):867-880. DOI: 10.1007/s11738-012-1130-8
2. Barbosa da Sliva, F. S.; Maia, L. C. Mycorrhization and phosphorus may be an alternative for increasing the production of metabolites in Myracrodruon urundeuva. Theor. Exp. Plant Physiol. 2018, 30:297-302. DOI: 10.1007/s40626-018-0123-4
3. Chen, M.; Yang, G.; Liu, D.; Li, M.; Qiu, H.; Guo, L.; Huang, L.; Chao, Z. Inoculation with Glomus mosseae Improves the Growth and Salvianolic Acid B Accumulation of Continuously Cropped Salvia miltiorrhiza. Appl. Sci. 2017, 7(7):692. DOI:10.3390/app7070692
4. Chen, M.; Yang, G.; Sheng, Y.; Li, P.; Qiu, H.; Zhou, X.; Huang, L.; Chao, Z. Glomus mosseae inoculation improves the root system architecture, photosynthetic efficiency and flavonoids accumulation of Liquorice under nutrient Stress. Front Plant Sci. 2017, 8:931. DOI: 10.3389/fpls.2017.00931
5. de Oliveira, P. T. F.; Santos, E. L. D.; da Sliva, W. A. V.; Ferreira, M. R. A.; Soares, L. A. L.; da Silva, F. A.; da Silva, F. S. B. Production of biomolecules of interest to the anxiolytic herbal medicine industry in yellow passionfruit leaves (Passiflora edulis f. flavicarpa) promoted by mycorrhizal inoculation. J Sci Food Agric. 2019, 99(7):3716-3720. DOI:10.1002/jsfa.9598
6. Duc, N. H.; Vo, A. T.; Haddidi, I.; Daood, H.; Posta, K. Arbuscular mycorrhizal fungi improve tolerance of the medicinal plant Eclipta prostrata (L.) and induce major changes in polyphenol profiles under salt stresses. Front Plant Sci. 2021, 11:612299. DOI: 10.3389/fpls.2020.612299

7. Fontana, A.; Reichelt, M.; Hempel, S.; Gershenzon, J.; Unsicker, S. The Effects of Arbuscular Mycorrhizal Fungi on Direct and Indirect Defense Metabolites of Plantago lanceolata L. J. Chem. Ecol. 2009, 35: 833-843. DOI: 10.1007/s10886-009-9654-0
8. Hristozkova, M,; Geneva, M.; Stancheva, I.; Boychinova, M.; Djonova, E. Contribution of arbuscular mycorrhizal fungi in attenuation of heavy metal impact on Calendula officinalis development. Appl Soil Ecol. 2016, 101:57-63. DOI: 10.1016/j.apsoil.2016.01.008
9. Hristozkova, M.; Gigova, L.; Geneva, M.; Stancheva, I.; Velikova, V.; Marinova, G. Influence of Mycorrhizal Fungi and Microalgae Dual Inoculation on Basil Plants Performance. Gesunde Pflanz. 2018, 70:99-107. DOI: 10.1007/s10343-018-0420-5
10. Lima, C. S.; Santos, H. R. S.; Albuquerque, U. P. d.; Silva, F. S. B. d. Mycorrhizal symbiosis increase the level of total foliar phenols and tannins in Commiphora leptophloeos (Mart.) J.B. Gillett seedlings. Ind Crops Prod. 2017, 104:28-32. DOI: 10.1016/j.indcrop.2017.04.020
11. Mandal, S.; Upadhyay, S.; Wajid, S.; Ram, M.; Jain, D. C.; Singh, V. P.; Abdin, M. Z.; Kapoor, R.Arbuscular mycorrhiza increase artemisinin accumulation in Artemisia annua by higher expression of key biosynthesis genes via enhanced jasmonic acid levels. Mcorrhiza. 2014, 25(5):345-57. DOI: 10.1007/s00572-014-0614-3.
12. Mandal, S.; Evelin, H.; Girl, B.; Singh, V. P.; Kapoor, R. Arbuscular mycorrhiza enhances the production of stevioside and rebaudioside-A in Stevia rebaudiana via nutritional and non-nutritional mechanisms. Appl Soil Ecol. 2013, 72:187-194. DOI: 10.1016/j.apsoil.2013.07.003
13. Merlin, E.; Melato, E.; Lourenco, E. L. B.; Jacomassi, E.; Junior, A. G.; Sete da Cruz, E.; Otenio, J. K.; da Sliva, C.; Alberton, O. Inoculation of arbuscular mycorrhizal fungi and phosphorus addition increase coarse mint (Plectranthus amboinicus Lour.) plant growth and essential oil content. Rhizosphere. 2020, 15: 100217. DOI: 10.1016/j.rhisph.2020.100217
14. Orujei, Y.; Shabani, L.; Sharifi Tehrani, M. Induction of glycyrrhizin and total phenolic compound production in licorice by using arbuscular mycorrhizal fungi. Russ. J. Plant Physiol. 2013, 60:855-960.DOI: 10.1134/S1021443713050129
15. Pistelli, L.; Ulivieri, V., Giovaneli, S.; Avio, L.; Giovannetti, M.; Pistelli, L. Arbuscular mycorrhizal fungi alter the content and composition of secondary metabolites in Bituminaria bituminosa L Plant Biol. 2017, 19(6):926-933. DOI:10.1111/plb.12608
16. Ran, Z.; Yang, X.; Zhang, Y.; Zhou, J.; Guo, L. Effects of arbuscular mycorrhizal fungi on photosynthesis and biosynthesis of ginsenoside in Panax quinquefolius L. Theor Exp Plant Physiol. 2021, 33(3):235-248. DOI: 10.1007/s40626-021-00208-y
17. Lazzara S.; Milltello, M.; Carrubba, A.; Napoli, E.; Saia, S. Arbuscular mycorrhizal fungi altered the hypericin, pseudohypericin, and hyperforin content in flowers of Hypericum perforatum grown under contrasting P availability in a highly organic substrate. Mycorrhiza. 2017, 27(4):345-354. DOI:10.1007/s00572-016-0756-6
18. Srivastava, S.; Conlan, X. A.; Cahill, D. M.; Adholeya, A. Rhizophagus irregularis as an elicitor of rosmarinic acid and antioxidant production by transformed roots of Ocimum basilicum in an in vitro co-culture system. Mycorrhiza. 2016, 26(8):919-930. DOI: 10.1007/s00572-016-0721-4
19. Tarraf, W.; Ruta, C.; Tagarelli, A.; De Cillis, F.; De Mastro, G. Influence of arbuscular mycorrhizae on plant growth, essential oil production and phosphorus uptake of

Salvia officinalis L. Ind Crops Prod. 2017, 102: 144-153. DOI:10.1016/j.indcrop.2017.03.010

20. Vieira, M. E.; Freitas, M. S. M.; Pecanha, D. A.; Lima, T. C.; Martins, M. A.; Vieira, I. J. C. Arbuscular mycorrhizal fungi and phosphorus in spilanthol and phenolic compound yield in jambu plants. Hortic Bras. 2021, 39(2):192-198. DOI: 10.1590/s0102-0536-20210210
21. Vo, A. T.; Haddidi, I.; Daood, H.; Mayer, Z.; Posta, K. Impact of arbuscular mycorrhizal inoculation and growth substrate on biomass and content of polyphenols in Eclipta prostrata. HortScience. 2019, 54(11):1976-1983. DOI: 10.21273/HORTSCI14227-19
22. Wu, Y. H.; Wang, H.; Liu, M.; Li, B.; Chen, X.; Ma, Y. T.; Yan, Z. Y. Effects of native arbuscular mycorrhizae isolated on root biomass and secondary metabolites of Salvia miltiorrhiza Bge. Front Plant Sci. 2021, 12:617892. DOI: 10.3389/fpls.2021.617892
23. Xie, W.; Hao, Z.; Yu, M.; Wu, Z.; Zhao, A.; Li, J.; Zhang, X.; Chen, B. Improved phosphorus nutrition by arbuscular mycorrhizal symbiosis as a key factor facilitating glycyrrhizin and liquiritin accumulation in Glycyrrhiza uralensis. Plant Soil. 2019, 439(1-2):243-257. DOI: 10.1007/s11104-018-3861-9
24. Xie, W.; Hao, Z.; Zhou, X.; Jiang, X.; Xu, L.; Wu, S.; Zhao, A.; Zhang, X.; Chen, B. Arbuscular mycorrhiza facilitates the accumulation of glycyrrhizin and liquiritin in Glycyrrhiza uralensis under drought stress. Mycorrhiza. 2018, 28(3):285-300. DOI: 10.1007/s00572-018-0827-y
25. Yang, X.; Ma, S.; Li, J. Effects of different soil remediation methods on inhibition of lead absorption and growth and quality of Dianthus superbus L.Environ.Sci. Pollut. Res. 2017, 24:28190-28196. DOI:10.1007/s11356-017-0089-9
26. Yang, Y.; Ou, X. H.; Yang, G.; Xia, Y. S.; Chen, M. L.; Guo, L. P.; Liu, D. H. Arbuscular mycorrhizal fungi regulate the growth and Phyto-Active compound of Salvia miltiorrhiza seedlings. Appl Sci. 2017, 7(1):68. DOI: 10.3390/app7010068
27. Yu, M.; Xie, W.; Zhang, X.; Zhang, S.; Wang, Y.; Hao, Z.; Chen, B. Arbuscular mycorrhizal fungi can compensate for the loss of indigenous microbial communities to support the growth of Liquorice (Glycyrrhiza uralensis Fisch.). Plants. 2019, 9(1):7. DOI: 10.3390/plants9010007
28. Zubek, S.; Mielcarek, S.; Turnau, K. Hypericin and pseudohypericin concentrations of a valuable medicinal plant Hypericum perforatum L. are enhanced by arbuscular mycorrhizal fungi. Mycorrhiza. 2012, 22(2):149-156. DOI: 10.1007/s00572-011-0391-1

Supplementary tables:

Table S1. Detailed information of medicinal active ingredients.

ID	reference	species	AMF inoculation	compounds	plant organs	n	X_C	X_T	S_C	S_T
1	Mandal et al., 2013	*Stevia rebaudiana*	S	terpenoids	A	5	2.1	4.64	0.21	0.464
2	Mandal et al., 2013	*Stevia rebaudiana*	S	terpenoids	A	5	2.9	4.28	0.29	0.428
3	Mandal et al., 2013	*Stevia rebaudiana*	S	terpenoids	A	5	2.16	3.09	0.216	0.309
4	Mandal et al., 2013	*Stevia rebaudiana*	S	terpenoids	A	5	2.3	3.56	0.23	0.356
5	Xie et al., 2018	*Glycyrrhiza uralensis*	S	terpenoids	B	5	3.00155	8.83101	0.44374769	2.828827238
6	Xie et al., 2018	*Glycyrrhiza uralensis*	S	terpenoids	B	5	3.5969	11.8078	0.388270844	2.329535619
7	Xie et al., 2018	*Glycyrrhiza uralensis*	S	terpenoids	B	5	4.3907	8.58295	0.554679022	1.164834892
8	Xie et al., 2018	*Glycyrrhiza uralensis*	S	flavonoids	B	5	1.27554	5.8452	0.221527255	0.636899242
9	Xie et al., 2018	*Glycyrrhiza uralensis*	S	flavonoids	B	5	0.780186	4.01238	0.249220956	0.775356571
10	Xie et al., 2018	*Glycyrrhiza uralensis*	S	flavonoids	B	5	0.705882	2.81115	0.138457329	0.91379154
11	Mandal et al., 2014	*Artemisia annua*	S	terpenoids	A	8	0.301699	0.491932	0.008917	0.010403
12	Mandal et al., 2014	*Artemisia annua*	S	terpenoids	A	8	0.271975	0.301699	0.01932	0.008917
13	Pistelli et al., 2017	*Bituminaria bituminosa*	S	others	A	6	0.18	0.01	0.07	0.004
14	Pistelli et al., 2017	*Bituminaria bituminosa*	S	others	A	6	1.4	0.16	0.11	0.07
15	Pistelli et al., 2017	*Bituminaria bituminosa*	S	others	A	6	0.00256	0.00295	0.00118	0.00042
16	Pistelli et al., 2017	*Bituminaria bituminosa*	S	others	A	6	0.00125	0.00007	0.00076	0.00003
17	Pistelli et al., 2017	*Bituminaria bituminosa*	S	others	A	6	0.00549	0.00993	0.00204	0.00019
18	Pistelli et al., 2017	*Bituminaria bituminosa*	S	others	A	6	0.00586	0.00075	0.00233	0.00025
19	Lazzara et al., 2017	*Hypericum perforatum*	M	phenols	A	4	2.80769	2.37821	0.42308	0.3205

20	Lazzara et al., 2017	*Hypericum perforatum*	M	phenols	A	4	2.26923	1.80769	0.4359	0.39744
21	Lazzara et al., 2017	*Hypericum perforatum*	M	flavonoids	A	4	0.0705882	0.189154	0.0099266	0.02978
22	Lazzara et al., 2017	*Hypericum perforatum*	M	flavonoids	A	4	0.230515	0.191912	0.050734	0.039706
23	Lazzara et al., 2017	*Hypericum perforatum*	M	flavonoids	A	4	0.104779	0.401654	0.020222	0.060662
24	Lazzara et al., 2017	*Hypericum perforatum*	M	flavonoids	A	4	0.431985	0.39614	0.080882	0.045956
25	Vieira et al., 2021	*Acmella oleacea*	S	alkaloids	A	4	0.27	2.03	0.027	0.203
26	Vieira et al., 2021	*Acmella oleacea*	S	alkaloids	A	4	0.76	2.68	0.076	0.268
27	Vieira et al., 2021	*Acmella oleacea*	S	alkaloids	A	4	4.4	3.7	0.44	0.37
28	Vieira et al., 2021	*Acmella oleacea*	S	alkaloids	A	4	0.27	1.09	0.027	0.109
29	Vieira et al., 2021	*Acmella oleacea*	S	alkaloids	A	4	0.76	5.2	0.076	0.52
30	Vieira et al., 2021	*Acmella oleacea*	S	alkaloids	A	4	4.4	5.2	0.44	0.52
31	Yu et al., 2019	*Glycyrrhiza uralensis*	S	terpenoids	B	4	11.9568	11.471	1.2956	0.8096
32	Yu et al., 2019	*Glycyrrhiza uralensis*	S	terpenoids	B	4	2.96896	12.3617	0.37786	3.2388
33	Yu et al., 2019	*Glycyrrhiza uralensis*	S	flavonoids	B	4	5.23243	4.51892	1.25406	1.27568
34	Yu et al., 2019	*Glycyrrhiza uralensis*	S	flavonoids	B	4	1.28649	5.95676	0.19458	2.33512
35	Yang et al., 2017a	*Salvia miltiorrhiza*	S	quinones	B	5	0.0524	0.1033	0.022	0.0325
36	Yang et al., 2017a	*Salvia miltiorrhiza*	S	quinones	B	5	0.0524	0.0781	0.022	0.0065
37	Yang et al., 2017a	*Salvia miltiorrhiza*	S	quinones	B	5	0.0524	0.0613	0.022	0.0024
38	Yang et al., 2017a	*Salvia miltiorrhiza*	S	quinones	B	5	0.0524	0.0817	0.022	0.022
39	Yang et al., 2017a	*Salvia miltiorrhiza*	S	quinones	B	5	0.1484	0.2502	0.0286	0.0393
40	Yang et al., 2017a	*Salvia miltiorrhiza*	S	quinones	B	5	0.1484	0.1339	0.0286	0.0356
41	Yang et al., 2017a	*Salvia miltiorrhiza*	S	quinones	B	5	0.1484	0.2914	0.0286	0.1079
42	Yang et al., 2017a	*Salvia miltiorrhiza*	S	quinones	B	5	0.1484	0.2618	0.0286	0.0716
43	Yang et al., 2017a	*Salvia miltiorrhiza*	S	quinones	B	5	0.0525	0.1002	0.0134	0.0194
44	Yang et al., 2017a	*Salvia miltiorrhiza*	S	quinones	B	5	0.0525	0.0382	0.0134	0.0043
45	Yang et al., 2017a	*Salvia miltiorrhiza*	S	quinones	B	5	0.0525	0.0812	0.0134	0.0043

46	Yang et al., 2017a	*Salvia miltiorrhiza*	S	quinones	B	5	0.0525	0.1133	0.0134	0.0256
47	Yang et al., 2017a	*Salvia miltiorrhiza*	S	quinones	B	5	0.2162	0.456	0.0677	0.0526
48	Yang et al., 2017a	*Salvia miltiorrhiza*	S	quinones	B	5	0.2162	0.3052	0.0677	0.0986
49	Yang et al., 2017a	*Salvia miltiorrhiza*	S	quinones	B	5	0.2162	0.2978	0.0677	0.03
50	Yang et al., 2017a	*Salvia miltiorrhiza*	S	quinones	B	5	0.2162	0.3743	0.0677	0.0319
51	Andrade et al., 2013	*Catharanthus roseus*	S	alkaloids	A	3	5.92095	4.52372	1.126179435	0.958447635
52	Andrade et al., 2013	*Catharanthus roseus*	S	alkaloids	A	3	0.249038	0.414423	0.074945838	0.05162897
53	Andrade et al., 2013	*Catharanthus roseus*	S	alkaloids	A	3	0.0697297	0.0940541	0.022068579	0.006821162
54	Andrade et al., 2013	*Catharanthus roseus*	S	alkaloids	A	3	4.29159	6.10093	0.569792754	1.03600887
55	Andrade et al., 2013	*Catharanthus roseus*	S	alkaloids	A	3	1.06415	2.26415	0.03921363	0.156871842
56	Andrade et al., 2013	*Catharanthus roseus*	S	others	A	3	16.0973	19.7042	3.470683408	2.082444686
57	Andrade et al., 2013	*Catharanthus roseus*	S	alkaloids	A	3	2.8083	2.29644	1.174105281	0.527149663
58	Andrade et al., 2013	*Catharanthus roseus*	S	alkaloids	A	3	0.245192	0.276923	0.0083277	0.019986134
59	Andrade et al., 2013	*Catharanthus roseus*	S	alkaloids	A	3	0.0664865	0.0732046	0.001203775	0.00561756
60	Andrade et al., 2013	*Catharanthus roseus*	S	alkaloids	A	3	2.42243	3.61869	0.414393156	1.476296185
61	Andrade et al., 2013	*Catharanthus roseus*	S	alkaloids	A	3	0.407547	0.362264	0.039217094	0.091504244
62	Andrade et al., 2013	*Catharanthus roseus*	S	others	A	3	6.87977	7.21374	0.925521349	1.156906016
63	Andrade et al., 2013	*Catharanthus roseus*	S	alkaloids	B	3	2.79626	1.98879	0.336693356	0.258993557
64	Andrade et al., 2013	*Catharanthus roseus*	S	alkaloids	B	3	3.35094	2.87547	0.352957314	0.352957314
65	Andrade et al., 2013	*Catharanthus roseus*	S	others	B	3	28.7882	25.7824	3.123580426	1.504112921
66	Hristozkova et al., 2016	*Calendula officinalis*	S	phenols	A	4	15.0741	16.963	2.8888	2.8148
67	Hristozkova et al., 2016	*Calendula officinalis*	S	phenols	A	4	15.0741	19.963	2.8888	2.7406
68	Hristozkova et al., 2016	*Calendula officinalis*	S	phenols	A	4	15.0741	17.3704	2.8888	2.8888
69	Hristozkova et al., 2016	*Calendula officinalis*	S	flavonoids	A	4	4.52804	5.48564	1.34064	1.39534
70	Hristozkova et al., 2016	*Calendula officinalis*	S	flavonoids	A	4	4.52804	9.12449	1.34064	1.50478
71	Hristozkova et al., 2016	*Calendula officinalis*	S	flavonoids	A	4	4.52804	6.44323	1.34064	1.34062

72	Yang at al., 2017b	*Dianthus superbus*	S	terpenoids	A	3	3.21622	6.51843	0.321622	0.651843
73	Wu et al., 2021	*Salvia miltiorrhiza*	S	phenols	B	6	0.12	0.14	0.02	0.03
74	Wu et al., 2021	*Salvia miltiorrhiza*	S	phenols	B	6	0.12	0.11	0.02	0.02
75	Wu et al., 2021	*Salvia miltiorrhiza*	S	phenols	B	6	0.12	0.13	0.02	0.03
76	Wu et al., 2021	*Salvia miltiorrhiza*	S	phenols	B	6	0.12	0.05	0.02	0
77	Wu et al., 2021	*Salvia miltiorrhiza*	S	phenols	B	6	0.12	0.08	0.02	0.02
78	Wu et al., 2021	*Salvia miltiorrhiza*	S	phenols	B	6	0.12	0.11	0.02	0.05
79	Wu et al., 2021	*Salvia miltiorrhiza*	S	phenols	B	6	0.12	0.15	0.02	0.05
80	Wu et al., 2021	*Salvia miltiorrhiza*	S	phenols	B	6	0.12	0.09	0.02	0.01
81	Wu et al., 2021	*Salvia miltiorrhiza*	S	organic acids	B	6	0.04	0.06	0.02	0.03
82	Wu et al., 2021	*Salvia miltiorrhiza*	S	organic acids	B	6	0.04	0.09	0.02	0.03
83	Wu et al., 2021	*Salvia miltiorrhiza*	S	organic acids	B	6	0.04	0.04	0.02	0
84	Wu et al., 2021	*Salvia miltiorrhiza*	S	organic acids	B	6	0.04	0.06	0.02	0.02
85	Wu et al., 2021	*Salvia miltiorrhiza*	S	organic acids	B	6	0.04	0.04	0.02	0
86	Wu et al., 2021	*Salvia miltiorrhiza*	S	organic acids	B	6	0.04	0.04	0.02	0.01
87	Wu et al., 2021	*Salvia miltiorrhiza*	S	organic acids	B	6	0.04	0.07	0.02	0.03
88	Wu et al., 2021	*Salvia miltiorrhiza*	S	organic acids	B	6	0.04	0.02	0.02	0.01
89	Wu et al., 2021	*Salvia miltiorrhiza*	S	phenols	B	6	3.34	4.15	0.75	0.5
90	Wu et al., 2021	*Salvia miltiorrhiza*	S	phenols	B	6	3.34	3.07	0.75	1.08
91	Wu et al., 2021	*Salvia miltiorrhiza*	S	phenols	B	6	3.34	3.22	0.75	1.01
92	Wu et al., 2021	*Salvia miltiorrhiza*	S	phenols	B	6	3.34	1.03	0.75	0.55
93	Wu et al., 2021	*Salvia miltiorrhiza*	S	phenols	B	6	3.34	3.31	0.75	0.68
94	Wu et al., 2021	*Salvia miltiorrhiza*	S	phenols	B	6	3.34	2.44	0.75	0.54
95	Wu et al., 2021	*Salvia miltiorrhiza*	S	phenols	B	6	3.34	4.76	0.75	0.42
96	Wu et al., 2021	*Salvia miltiorrhiza*	S	phenols	B	6	3.34	2.73	0.75	0.19
97	Wu et al., 2021	*Salvia miltiorrhiza*	S	phenols	B	6	30.75	45.01	3	3.9

98	Wu et al., 2021	*Salvia miltiorrhiza*	S	phenols	B	6	30.75	33.7	3	6.91
99	Wu et al., 2021	*Salvia miltiorrhiza*	S	phenols	B	6	30.75	36.4	3	4.81
100	Wu et al., 2021	*Salvia miltiorrhiza*	S	phenols	B	6	30.75	18.41	3	6.35
101	Wu et al., 2021	*Salvia miltiorrhiza*	S	phenols	B	6	30.75	38.9	3	5.86
102	Wu et al., 2021	*Salvia miltiorrhiza*	S	phenols	B	6	30.75	36.64	3	2.01
103	Wu et al., 2021	*Salvia miltiorrhiza*	S	phenols	B	6	30.75	41.23	3	8.27
104	Wu et al., 2021	*Salvia miltiorrhiza*	S	phenols	B	6	30.75	30.23	3	2.35
105	Wu et al., 2021	*Salvia miltiorrhiza*	S	phenols	B	6	0.31	0.44	0.03	0.03
106	Wu et al., 2021	*Salvia miltiorrhiza*	S	phenols	B	6	0.31	0.37	0.03	0.07
107	Wu et al., 2021	*Salvia miltiorrhiza*	S	phenols	B	6	0.31	0.33	0.03	0.04
108	Wu et al., 2021	*Salvia miltiorrhiza*	S	phenols	B	6	0.31	0.13	0.03	0.07
109	Wu et al., 2021	*Salvia miltiorrhiza*	S	phenols	B	6	0.31	0.33	0.03	0.07
110	Wu et al., 2021	*Salvia miltiorrhiza*	S	phenols	B	6	0.31	0.3	0.03	0.05
111	Wu et al., 2021	*Salvia miltiorrhiza*	S	phenols	B	6	0.31	0.45	0.03	0.12
112	Wu et al., 2021	*Salvia miltiorrhiza*	S	phenols	B	6	0.31	0.36	0.03	0.04
113	Wu et al., 2021	*Salvia miltiorrhiza*	S	quinones	B	6	0.05	0.05	0.02	0.03
114	Wu et al., 2021	*Salvia miltiorrhiza*	S	quinones	B	6	0.05	0.12	0.02	0.04
115	Wu et al., 2021	*Salvia miltiorrhiza*	S	quinones	B	6	0.05	0.04	0.02	0.02
116	Wu et al., 2021	*Salvia miltiorrhiza*	S	quinones	B	6	0.05	0.06	0.02	0.02
117	Wu et al., 2021	*Salvia miltiorrhiza*	S	quinones	B	6	0.05	0.03	0.02	0.01
118	Wu et al., 2021	*Salvia miltiorrhiza*	S	quinones	B	6	0.05	0.03	0.02	0
119	Wu et al., 2021	*Salvia miltiorrhiza*	S	quinones	B	6	0.05	0.06	0.02	0.01
120	Wu et al., 2021	*Salvia miltiorrhiza*	S	quinones	B	6	0.05	0.06	0.02	0.02
121	Wu et al., 2021	*Salvia miltiorrhiza*	S	quinones	B	6	0.007	0.009	0.003	0.002
122	Wu et al., 2021	*Salvia miltiorrhiza*	S	quinones	B	6	0.007	0.007	0.003	0.003
123	Wu et al., 2021	*Salvia miltiorrhiza*	S	quinones	B	6	0.007	0.008	0.003	0.006

124	Wu et al., 2021	*Salvia miltiorrhiza*	S	quinones	B	6	0.007	0.011	0.003	0.001
125	Wu et al., 2021	*Salvia miltiorrhiza*	S	quinones	B	6	0.007	0.008	0.003	0.001
126	Wu et al., 2021	*Salvia miltiorrhiza*	S	quinones	B	6	0.007	0.007	0.003	0.003
127	Wu et al., 2021	*Salvia miltiorrhiza*	S	quinones	B	6	0.007	0.013	0.003	0.002
128	Wu et al., 2021	*Salvia miltiorrhiza*	S	quinones	B	6	0.007	0.011	0.003	0.003
129	Wu et al., 2021	*Salvia miltiorrhiza*	S	quinones	B	6	0.94	0.88	0.59	0.62
130	Wu et al., 2021	*Salvia miltiorrhiza*	S	quinones	B	6	0.94	1.74	0.59	0.84
131	Wu et al., 2021	*Salvia miltiorrhiza*	S	quinones	B	6	0.94	1.04	0.59	1.05
132	Wu et al., 2021	*Salvia miltiorrhiza*	S	quinones	B	6	0.94	1.33	0.59	0.3
133	Wu et al., 2021	*Salvia miltiorrhiza*	S	quinones	B	6	0.94	0.52	0.59	0.12
134	Wu et al., 2021	*Salvia miltiorrhiza*	S	quinones	B	6	0.94	0.77	0.59	0.24
135	Wu et al., 2021	*Salvia miltiorrhiza*	S	quinones	B	6	0.94	0.71	0.59	0.14
136	Wu et al., 2021	*Salvia miltiorrhiza*	S	quinones	B	6	0.94	1.17	0.59	0.31
137	Wu et al., 2021	*Salvia miltiorrhiza*	S	quinones	B	6	0.53	0.59	0.47	0.33
138	Wu et al., 2021	*Salvia miltiorrhiza*	S	quinones	B	6	0.53	0.76	0.47	0.31
139	Wu et al., 2021	*Salvia miltiorrhiza*	S	quinones	B	6	0.53	0.59	0.47	0.47
140	Wu et al., 2021	*Salvia miltiorrhiza*	S	quinones	B	6	0.53	0.95	0.47	0.18
141	Wu et al., 2021	*Salvia miltiorrhiza*	S	quinones	B	6	0.53	0.41	0.47	0.09
142	Wu et al., 2021	*Salvia miltiorrhiza*	S	quinones	B	6	0.53	0.53	0.47	0.18
143	Wu et al., 2021	*Salvia miltiorrhiza*	S	quinones	B	6	0.53	0.43	0.47	0.13
144	Wu et al., 2021	*Salvia miltiorrhiza*	S	quinones	B	6	0.53	0.64	0.47	0.19
145	Chen et al., 2017a	*Glycyrrhiza uralensis*	S	terpenoids	B	6	4.8	12.4	0.48	1.24
146	Chen et al., 2017a	*Glycyrrhiza uralensis*	S	terpenoids	B	6	1.37	3.8	0.137	0.38
147	Chen et al., 2017a	*Glycyrrhiza uralensis*	S	flavonoids	B	6	0.65	3.76	0.065	0.376
148	Chen et al., 2017a	*Glycyrrhiza uralensis*	S	flavonoids	B	6	0.085	0.47	0.0085	0.047
149	Chen et al., 2017a	*Glycyrrhiza uralensis*	S	terpenoids	B	6	0.12	0.91	0.012	0.091

150	Chen et al., 2017a	*Glycyrrhiza uralensis*	S	flavonoids	B	6	0.033	0.18	0.0033	0.018
151	Zubek et al., 2012	*Hypericum perforatum*	S	flavonoids	A	14	0.0306646	0.0504482	0.0081607	0.0114992
152	Zubek et al., 2012	*Hypericum perforatum*	S	flavonoids	A	14	0.0306646	0.0294281	0.0081607	0.0096445
153	Zubek et al., 2012	*Hypericum perforatum*	M	flavonoids	A	14	0.0306646	0.0490881	0.0081607	0.0091499
154	Zubek et al., 2012	*Hypericum perforatum*	S	flavonoids	A	14	0.0533232	0.0737178	0.0133839	0.0172078
155	Zubek et al., 2012	*Hypericum perforatum*	S	flavonoids	A	14	0.0533232	0.0545979	0.0133839	0.0144461
156	Zubek et al., 2012	*Hypericum perforatum*	M	flavonoids	A	14	0.0533232	0.0843399	0.0133839	0.0327161
157	Zubek et al., 2012	*Hypericum perforatum*	S	phenols	A	14	2.67577	2.57338	0.45734	0.64164
158	Zubek et al., 2012	*Hypericum perforatum*	S	phenols	A	14	2.67577	2.47099	0.45734	0.51877
159	Zubek et al., 2012	*Hypericum perforatum*	M	phenols	A	14	2.67577	2.79181	0.45734	0.68259
160	Vo et al., 2019	*Eclipta prostrata*	M	others	A	3	2.37625	3.26399	0.6041	0.95064
161	Vo et al., 2019	*Eclipta prostrata*	M	others	A	3	1.9538	1.36273	0.40792	0.07905
162	Vo et al., 2019	*Eclipta prostrata*	M	others	A	3	2.58827	3.06397	0.39936	0.52418
163	Vo et al., 2019	*Eclipta prostrata*	M	others	A	3	0.95739	1.01037	0.12932	0.14122
164	Vo et al., 2019	*Eclipta prostrata*	M	others	A	3	0.65931	0.75771	0.08617	0.11333
165	Vo et al., 2019	*Eclipta prostrata*	M	others	A	3	1.16519	1.18825	0.0993	0.09034
166	Vo et al., 2019	*Eclipta prostrata*	M	phenols	A	3	11.19	14.3559	0.6004	3.821
167	Vo et al., 2019	*Eclipta prostrata*	M	phenols	A	3	13.155	13.3188	3.7664	2.0742
168	Vo et al., 2019	*Eclipta prostrata*	M	phenols	A	3	18.6681	19.7598	2.4018	1.6922
169	Vo et al., 2019	*Eclipta prostrata*	M	phenols	A	3	8.0786	9.06114	2.4017	2.40176
170	Vo et al., 2019	*Eclipta prostrata*	M	phenols	A	3	4.47598	4.85808	1.74673	2.01965
171	Vo et al., 2019	*Eclipta prostrata*	M	phenols	A	3	3.82096	5.84061	1.03712	1.8559
172	Xie et al., 2019	*Glycyrrhiza uralensis*	S	terpenoids	B	4	1.2869	5.94362	0.1592	2.30844
173	Xie et al., 2019	*Glycyrrhiza uralensis*	S	flavonoids	B	4	1.14096	13.1343	0.42454	3.7148
174	Orujei et al., 2013	*Glycyrrhiza glabra*	S	terpenoids	B	3	0.95	4.03	0.095	0.403
175	Orujei et al., 2013	*Glycyrrhiza glabra*	S	terpenoids	B	3	0.95	3.12	0.095	0.312

176	Orujei et al., 2013	*Glycyrrhiza glabra*	S	terpenoids	B	3	0.22	2.1	0.022	0.21
177	Orujei et al., 2013	*Glycyrrhiza glabra*	S	terpenoids	B	3	0.22	0.69	0.022	0.069
178	Tarraf et al., 2017	*Salvia officinalis*	S	terpenoids	A	7	1.06	0.88	0.07	0.17
179	Tarraf et al., 2017	*Salvia officinalis*	S	terpenoids	A	7	1.06	1.03	0.07	0.07
180	Tarraf et al., 2017	*Salvia officinalis*	S	terpenoids	A	7	1.06	0.99	0.07	0.2
181	Hristozkova et al., 2018	*Ocimum basilicum*	S	flavonoids	A	9	0.519	0.537	0.0519	0.0537
182	Merlin et al., 2020	*Plectranthus amboinicus*	S	terpenoids	A	8	0.230675	0.611861	0.064782295	0.171210351
183	Merlin et al., 2020	*Plectranthus amboinicus*	S	terpenoids	A	8	0.230675	0.171779	0.064782295	0.050900375
184	Merlin et al., 2020	*Plectranthus amboinicus*	S	terpenoids	A	8	0.183231	0.471166	0.046273068	0.134189068
185	Merlin et al., 2020	*Plectranthus amboinicus*	S	terpenoids	A	8	0.183231	0.171779	0.046273068	0.050900375
186	Chen et al., 2017b	*Salvia miltiorrhiza*	S	phenols	A	3	0.89	0.63	0.034641016	0.155884573
187	Chen et al., 2017b	*Salvia miltiorrhiza*	S	phenols	A	3	1.14	1.39	0.034641016	0.155884573
188	Chen et al., 2017b	*Salvia miltiorrhiza*	S	phenols	B	3	0.33	0.34	0.051961524	0.034641016
189	Chen et al., 2017b	*Salvia miltiorrhiza*	S	phenols	B	3	0.76	0.83	0	0.017320508
190	Lima et al., 2017	*Commiphora leptophloeos*	S	phenols	A	5	1.57	1.97	0.44	0.44
191	Lima et al., 2017	*Commiphora leptophloeos*	S	phenols	A	5	1.57	2.24	0.44	0.44
192	Lima et al., 2017	*Commiphora leptophloeos*	S	phenols	A	5	1.57	2.29	0.44	0.44
193	Lima et al., 2017	*Commiphora leptophloeos*	S	flavonoids	A	5	0.04988	0.04306	0.01333	0.01333
194	Lima et al., 2017	*Commiphora leptophloeos*	S	flavonoids	A	5	0.04988	0.05051	0.01333	0.01333
195	Lima et al., 2017	*Commiphora leptophloeos*	S	flavonoids	A	5	0.04988	0.0513	0.01333	0.01333
196	Lima et al., 2017	*Commiphora leptophloeos*	S	phenols	A	5	1.41	1.89	0.47	0.47
197	Lima et al., 2017	*Commiphora leptophloeos*	S	phenols	A	5	1.41	2.19	0.47	0.47
198	Lima et al., 2017	*Commiphora leptophloeos*	S	phenols	A	5	1.41	2.29	0.47	0.47
199	Barbosa da Silva et al., 2018	*Myracrodruon urundeuva*	M	phenols	A	5	26.41	26.01	2.641	2.601
200	Barbosa da Silva et al., 2018	*Myracrodruon urundeuva*	M	phenols	A	5	27.84	49.84	2.784	4.984
201	Barbosa da Silva et al., 2018	*Myracrodruon urundeuva*	M	phenols	A	5	28.89	21.37	2.889	2.137

202	Felix de Oliveira et al., 2019	*Passiflora edulis*	S	flavonoids	A	5	0.11	0.18	0.011	0.018
203	Felix de Oliveira et al., 2019	*Passiflora edulis*	S	flavonoids	A	5	0.11	0.17	0.011	0.017
204	Felix de Oliveira et al., 2019	*Passiflora edulis*	S	flavonoids	A	5	0.11	0.17	0.011	0.017
205	Srivastava et al., 2016	*Ocimum basilicum*	S	phenols	B	3	31.45	32.42	2.47	2.62
206	Srivastava et al., 2016	*Ocimum basilicum*	S	phenols	B	3	18.19	37.54	5.91	8.84
207	Srivastava et al., 2016	*Ocimum basilicum*	S	phenols	B	3	14.47	22.88	2.59	2.29
208	Srivastava et al., 2016	*Ocimum basilicum*	S	phenols	B	3	37.54	57.2	1.82	10.45
209	Srivastava et al., 2016	*Ocimum basilicum*	S	phenols	B	3	81.82	127.87	18.21	2.05
210	Srivastava et al., 2016	*Ocimum basilicum*	S	phenols	B	3	35.82	55.6	5.29	7.87
211	Srivastava et al., 2016	*Ocimum basilicum*	S	phenols	B	3	50.98	96.52	5.58	18.54
212	Srivastava et al., 2016	*Ocimum basilicum*	S	phenols	B	3	98.66	140.53	4.77	5.26
213	Srivastava et al., 2016	*Ocimum basilicum*	S	phenols	B	3	65.52	94.97	6.89	13.05
214	Fontana et al., 2009	*Plantago lanceolata*	S	terpenoids	A	29	3.03	2.79	1.723252738	1.723252738
215	Fontana et al., 2009	*Plantago lanceolata*	S	terpenoids	A	29	2.95	2.87	1.777104386	1.884807682
216	Fontana et al., 2009	*Plantago lanceolata*	S	terpenoids	A	29	2.94	3.26	2.531027459	1.830956034
217	Fontana et al., 2009	*Plantago lanceolata*	S	terpenoids	A	29	7.35	7.13	2.746434052	3.123395588
218	Fontana et al., 2009	*Plantago lanceolata*	S	terpenoids	A	29	6.54	6.61	3.231098884	2.907988996
219	Fontana et al., 2009	*Plantago lanceolata*	S	terpenoids	A	29	5.37	6.89	2.907988996	2.638730755
220	Duc et al., 2021	*Eclipta prostrata*	M	phenols	A	3	1.65333	3.91111	0.55111	0.72889
221	Duc et al., 2021	*Eclipta prostrata*	M	phenols	A	3	4.35556	2.70222	0.46222	0.65778
222	Duc et al., 2021	*Eclipta prostrata*	M	phenols	A	3	1.04889	1.92	0.19555	0.05333
223	Duc et al., 2021	*Eclipta prostrata*	M	phenols	A	3	6.13333	6.88	1.12	0.40889
224	Duc et al., 2021	*Eclipta prostrata*	M	phenols	A	3	1.92	3.21778	0.19556	0.67555
225	Duc et al., 2021	*Eclipta prostrata*	M	phenols	A	3	1.12	6.06222	0.17778	0.24889
226	Ran et al., 2021	*Panax quinquefolius*	S	terpenoids	B	6	0.66004	0.823062	0.005964	0.033797
227	Ran et al., 2021	*Panax quinquefolius*	S	terpenoids	B	6	0.66004	0.779324	0.005964	0.04175

228	Ran et al., 2021	*Panax quinquefolius*	S	terpenoids	B	6	0.701789	0.858847	0.023857	0.06163
229	Ran et al., 2021	*Panax quinquefolius*	S	terpenoids	B	6	0.701789	0.831014	0.023857	0.047714
230	Ran et al., 2021	*Panax quinquefolius*	S	terpenoids	B	6	0.0755467	0.0755467	0.0079523	0.0019881
231	Ran et al., 2021	*Panax quinquefolius*	S	terpenoids	B	6	0.0755467	0.0874751	0.0079523	0.0039762
232	Ran et al., 2021	*Panax quinquefolius*	S	terpenoids	B	6	0.083499	0.101392	0.0059642	0.00994
233	Ran et al., 2021	*Panax quinquefolius*	S	terpenoids	B	6	0.083499	0.0854871	0.0059642	0.001988

Note: S: Single AMF inoculation; M: Multi-AMF inoculation; A: Aboveground organs; B: Belowground organs.

Table S2. Detailed information of physiological variables.

ID	reference	species	AMF inoculation	compounds	plant organs	n	physiological factor	X_C	X_T	S_C	S_T
1	Mandal et al., 2013	*Stevia rebaudiana*	S	terpenoids	A	5	Chl-A	1.60985	1.92708	0.00947	0.05209
2	Mandal et al., 2013	*Stevia rebaudiana*	S	terpenoids	A	5	Chl-A	1.70928	1.82292	0.00474	0.01421
3	Mandal et al., 2013	*Stevia rebaudiana*	S	terpenoids	A	5	Chl-A	1.60985	1.92708	0.00947	0.05209
4	Mandal et al., 2013	*Stevia rebaudiana*	S	terpenoids	A	5	Chl-A	1.70928	1.82292	0.00474	0.01421
33	Yu et al., 2019	*Glycyrrhiza uralensis*	S	terpenoids	B	4	Chl-A	1.32423	1.39249	0.314	0.09556
34	Yu et al., 2019	*Glycyrrhiza uralensis*	S	terpenoids	B	4	Chl-A	0.382253	1.39249	0.04778	0.17748
35	Yu et al., 2019	*Glycyrrhiza uralensis*	S	flavonoids	B	4	Chl-A	1.32423	1.39249	0.314	0.09556
36	Yu et al., 2019	*Glycyrrhiza uralensis*	S	flavonoids	B	4	Chl-A	0.382253	1.39249	0.04778	0.17748
68	Felix de Oliveira et al., 2019	*Passiflora edulis*	S	flavonoids	A	5	Chl-A	30.68	31.83	3.068	3.183
69	Felix de Oliveira et al., 2019	*Passiflora edulis*	S	flavonoids	A	5	Chl-A	30.68	34.05	3.068	3.405
70	Felix de Oliveira et al., 2019	*Passiflora edulis*	S	flavonoids	A	5	Chl-A	30.68	33.8	3.068	3.38

82	Ran et al., 2021	*Panax quinquefolius*	S	terpenoids	B	6	Chl-A	7.10204	9.08571	0.19592	0.44082
83	Ran et al., 2021	*Panax quinquefolius*	S	terpenoids	B	6	Chl-A	7.10204	9.23265	0.19592	0.34286
84	Ran et al., 2021	*Panax quinquefolius*	S	terpenoids	B	6	Chl-A	7.10204	9.08571	0.19592	0.44082
85	Ran et al., 2021	*Panax quinquefolius*	S	terpenoids	B	6	Chl-A	7.10204	9.23265	0.19592	0.34286
86	Ran et al., 2021	*Panax quinquefolius*	S	terpenoids	B	6	Chl-A	7.10204	9.08571	0.19592	0.44082
87	Ran et al., 2021	*Panax quinquefolius*	S	terpenoids	B	6	Chl-A	7.10204	9.23265	0.19592	0.34286
88	Ran et al., 2021	*Panax quinquefolius*	S	terpenoids	B	6	Chl-A	7.10204	9.08571	0.19592	0.44082
89	Ran et al., 2021	*Panax quinquefolius*	S	terpenoids	B	6	Chl-A	7.10204	9.23265	0.19592	0.34286
5	Mandal et al., 2013	*Stevia rebaudiana*	S	terpenoids	A	5	Chl-B	0.227273	1.47727	0.009469	0.00474
6	Mandal et al., 2013	*Stevia rebaudiana*	S	terpenoids	A	5	Chl-B	0.677083	1.13163	0.142046	0.02841
7	Mandal et al., 2013	*Stevia rebaudiana*	S	terpenoids	A	5	Chl-B	0.227273	1.47727	0.009469	0.00474
8	Mandal et al., 2013	*Stevia rebaudiana*	S	terpenoids	A	5	Chl-B	0.677083	1.13163	0.142046	0.02841
37	Yu et al., 2019	*Glycyrrhiza uralensis*	S	terpenoids	B	4	Chl-B	2.86449	2.9331	0.89194	0.2573
38	Yu et al., 2019	*Glycyrrhiza uralensis*	S	terpenoids	B	4	Chl-B	1.37221	3.06175	0.10292	0.58318
39	Yu et al., 2019	*Glycyrrhiza uralensis*	S	flavonoids	B	4	Chl-B	2.86449	2.9331	0.89194	0.2573
40	Yu et al., 2019	*Glycyrrhiza uralensis*	S	flavonoids	B	4	Chl-B	1.37221	3.06175	0.10292	0.58318
71	Felix de Oliveira et al., 2019	*Passiflora edulis*	S	flavonoids	A	5	Chl-B	9.3	9.57	0.93	0.957
72	Felix de Oliveira et al., 2019	*Passiflora edulis*	S	flavonoids	A	5	Chl-B	9.3	12.07	0.93	1.207
73	Felix de Oliveira et al., 2019	*Passiflora edulis*	S	flavonoids	A	5	Chl-B	9.3	12.65	0.93	1.265
90	Ran et al., 2021	*Panax quinquefolius*	S	terpenoids	B	6	Chl-B	3.94286	4.43265	0.07347	0.09796
91	Ran et al., 2021	*Panax quinquefolius*	S	terpenoids	B	6	Chl-B	3.94286	4.3102	0.07347	0.12245
92	Ran et al., 2021	*Panax quinquefolius*	S	terpenoids	B	6	Chl-B	3.94286	4.43265	0.07347	0.09796
93	Ran et al., 2021	*Panax quinquefolius*	S	terpenoids	B	6	Chl-B	3.94286	4.3102	0.07347	0.12245

94	Ran et al., 2021	*Panax quinquefolius*	S	terpenoids	B	6	Chl-B	3.94286	4.43265	0.07347	0.09796
95	Ran et al., 2021	*Panax quinquefolius*	S	terpenoids	B	6	Chl-B	3.94286	4.3102	0.07347	0.12245
96	Ran et al., 2021	*Panax quinquefolius*	S	terpenoids	B	6	Chl-B	3.94286	4.43265	0.07347	0.09796
97	Ran et al., 2021	*Panax quinquefolius*	S	terpenoids	B	6	Chl-B	3.94286	4.3102	0.07347	0.12245
56	Lima et al., 2017	*Commiphora leptophloeos*	S	phenols	A	5	CHO	359.01	147.43	97.92	97.92
57	Lima et al., 2017	*Commiphora leptophloeos*	S	phenols	A	5	CHO	359.01	224.68	97.92	97.92
58	Lima et al., 2017	*Commiphora leptophloeos*	S	phenols	A	5	CHO	359.01	160.29	97.92	97.92
59	Lima et al., 2017	*Commiphora leptophloeos*	S	flavonoids	A	5	CHO	359.01	147.43	97.92	97.92
60	Lima et al., 2017	*Commiphora leptophloeos*	S	flavonoids	A	5	CHO	359.01	224.68	97.92	97.92
61	Lima et al., 2017	*Commiphora leptophloeos*	S	flavonoids	A	5	CHO	359.01	160.29	97.92	97.92
62	Lima et al., 2017	*Commiphora leptophloeos*	S	phenols	A	5	CHO	359.01	147.43	97.92	97.92
63	Lima et al., 2017	*Commiphora leptophloeos*	S	phenols	A	5	CHO	359.01	224.68	97.92	97.92
64	Lima et al., 2017	*Commiphora leptophloeos*	S	phenols	A	5	CHO	359.01	160.29	97.92	97.92
15	Xie et al., 2018	*Glycyrrhiza uralensis*	S	terpenoids	B	5	Gs	1.50215	9.12898	0.419285106	0.545734751
16	Xie et al., 2018	*Glycyrrhiza uralensis*	S	terpenoids	B	5	Gs	1.55687	7.94242	0.545734751	1.387010606

17	Xie et al., 2018	*Glycyrrhiza uralensis*	S	terpenoids	B	5	Gs	0.74651	5.64615	0.209329504	3.153481947
18	Xie et al., 2018	*Glycyrrhiza uralensis*	S	flavonoids	B	5	Gs	1.50215	9.12898	0.419285106	0.545734751
19	Xie et al., 2018	*Glycyrrhiza uralensis*	S	flavonoids	B	5	Gs	1.55687	7.94242	0.545734751	1.387010606
20	Xie et al., 2018	*Glycyrrhiza uralensis*	S	flavonoids	B	5	Gs	0.74651	5.64615	0.209329504	3.153481947
45	Yu et al., 2019	*Glycyrrhiza uralensis*	S	terpenoids	B	4	Gs	0.0898547	0.10009	0.0400386	0.019494
46	Yu et al., 2019	*Glycyrrhiza uralensis*	S	terpenoids	B	4	Gs	0.0298815	0.119738	0.000014	0.02052
47	Yu et al., 2019	*Glycyrrhiza uralensis*	S	flavonoids	B	4	Gs	0.0898547	0.10009	0.0400386	0.019494
48	Yu et al., 2019	*Glycyrrhiza uralensis*	S	flavonoids	B	4	Gs	0.0298815	0.119738	0.000014	0.02052
51	Xie et al., 2019	*Glycyrrhiza uralensis*	S	terpenoids	B	4	Gs	0.0173494	0.11346	0.0077108	0.027538
52	Xie et al., 2019	*Glycyrrhiza uralensis*	S	flavonoids	B	4	Gs	0.0173494	0.11346	0.0077108	0.027538
54	Hristozkova et al., 2018	*Ocimum basilicum*	S	flavonoids	A	9	Gs	0.09	0.17	0.009	0.017
106	Ran et al., 2021	*Panax quinquefolius*	S	terpenoids	B	6	Gs	0.02	0.027	0.001	0.003
107	Ran et al., 2021	*Panax quinquefolius*	S	terpenoids	B	6	Gs	0.02	0.024	0.001	0.003
10	Ran et al., 2021	*Panax quinquefolius*	S	terpenoids	B	6	Gs	0.02	0.027	0.001	0.003

8											
109	Ran et al., 2021	*Panax quinquefolius*	S	terpenoids	B	6	Gs	0.02	0.024	0.001	0.003
110	Ran et al., 2021	*Panax quinquefolius*	S	terpenoids	B	6	Gs	0.02	0.027	0.001	0.003
111	Ran et al., 2021	*Panax quinquefolius*	S	terpenoids	B	6	Gs	0.02	0.024	0.001	0.003
112	Ran et al., 2021	*Panax quinquefolius*	S	terpenoids	B	6	Gs	0.02	0.027	0.001	0.003
113	Ran et al., 2021	*Panax quinquefolius*	S	terpenoids	B	6	Gs	0.02	0.024	0.001	0.003
9	Xie et al., 2018	*Glycyrrhiza uralensis*	S	terpenoids	B	5	Pn	0.0413793	0.137931	0.007009626	0.01822619
10	Xie et al., 2018	*Glycyrrhiza uralensis*	S	terpenoids	B	5	Pn	0.0341693	0.0896552	0.004906604	0.015421043
11	Xie et al., 2018	*Glycyrrhiza uralensis*	S	terpenoids	B	5	Pn	0.0275862	0.0611285	0.012617237	0.030141526
12	Xie et al., 2018	*Glycyrrhiza uralensis*	S	flavonoids	B	5	Pn	0.0413793	0.137931	0.007009626	0.01822619
13	Xie et al., 2018	*Glycyrrhiza uralensis*	S	flavonoids	B	5	Pn	0.0341693	0.0896552	0.004906604	0.015421043
14	Xie et al., 2018	*Glycyrrhiza uralensis*	S	flavonoids	B	5	Pn	0.0275862	0.0611285	0.012617237	0.030141526
41	Yu et al., 2019	*Glycyrrhiza uralensis*	S	terpenoids	B	4	Pn	9.32689	11.4396	3.09182	1.9066
42	Yu et al., 2019	*Glycyrrhiza uralensis*	S	terpenoids	B	4	Pn	1.18519	10.306	0.3607	1.4428
43	Yu et al., 2019	*Glycyrrhiza uralensis*	S	flavonoids	B	4	Pn	9.32689	11.4396	3.09182	1.9066

44	Yu et al., 2019	*Glycyrrhiza uralensis*	S	flavonoids	B	4	Pn	1.18519	10.306	0.3607	1.4428
49	Xie et al., 2019	*Glycyrrhiza uralensis*	S	terpenoids	B	4	Pn	1.21859	10.2857	0.33046	1.4872
50	Xie et al., 2019	*Glycyrrhiza uralensis*	S	flavonoids	B	4	Pn	1.21859	10.2857	0.33046	1.4872
53	Hristozkova et al., 2018	*Ocimum basilicum*	S	flavonoids	A	9	Pn	2.55	3.29	0.255	0.329
98	Ran et al., 2021	*Panax quinquefolius*	S	terpenoids	B	6	Pn	8.132	11.879	1.054	1.135
99	Ran et al., 2021	*Panax quinquefolius*	S	terpenoids	B	6	Pn	8.132	10.426	1.054	1.204
100	Ran et al., 2021	*Panax quinquefolius*	S	terpenoids	B	6	Pn	8.132	11.879	1.054	1.135
101	Ran et al., 2021	*Panax quinquefolius*	S	terpenoids	B	6	Pn	8.132	10.426	1.054	1.204
102	Ran et al., 2021	*Panax quinquefolius*	S	terpenoids	B	6	Pn	8.132	11.879	1.054	1.135
103	Ran et al., 2021	*Panax quinquefolius*	S	terpenoids	B	6	Pn	8.132	10.426	1.054	1.204
104	Ran et al., 2021	*Panax quinquefolius*	S	terpenoids	B	6	Pn	8.132	11.879	1.054	1.135
105	Ran et al., 2021	*Panax quinquefolius*	S	terpenoids	B	6	Pn	8.132	10.426	1.054	1.204
27	Pistelli et al., 2017	*Bituminaria bituminosa*	S	others	A	6	TC	88.54	123.6	18.63	8.64
28	Pistelli et al., 2017	*Bituminaria bituminosa*	S	others	A	6	TC	88.54	123.6	18.63	8.64
29	Pistelli et al., 2017	*Bituminaria bituminosa*	S	others	A	6	TC	98.07	85.52	9.807	8.552
30	Pistelli et al., 2017	*Bituminaria bituminosa*	S	others	A	6	TC	88.54	123.6	18.63	8.64
31	Pistelli et al., 2017	*Bituminaria bituminosa*	S	others	A	6	TC	98.07	85.52	9.807	8.552
32	Pistelli et al., 2017	*Bituminaria bituminosa*	S	others	A	6	TC	88.54	123.6	18.63	8.64
65	Felix de Oliveira et al., 2019	*Passiflora edulis*	S	flavonoids	A	5	TC	39.98	41.4	3.998	4.14

66	Felix de Oliveira et al., 2019	*Passiflora edulis*	S	flavonoids	A	5	TC	39.98	46.12	3.998	4.612
67	Felix de Oliveira et al., 2019	*Passiflora edulis*	S	flavonoids	A	5	TC	39.98	46.45	3.998	4.645
74	Ran et al., 2021	*Panax quinquefolius*	S	terpenoids	B	6	TC	1.78776	2.00816	0.04897	0.09796
75	Ran et al., 2021	*Panax quinquefolius*	S	terpenoids	B	6	TC	1.78776	1.73878	0.04897	0.04898
76	Ran et al., 2021	*Panax quinquefolius*	S	terpenoids	B	6	TC	1.78776	2.00816	0.04897	0.09796
77	Ran et al., 2021	*Panax quinquefolius*	S	terpenoids	B	6	TC	1.78776	1.73878	0.04897	0.04898
78	Ran et al., 2021	*Panax quinquefolius*	S	terpenoids	B	6	TC	1.78776	2.00816	0.04897	0.09796
79	Ran et al., 2021	*Panax quinquefolius*	S	terpenoids	B	6	TC	1.78776	1.73878	0.04897	0.04898
80	Ran et al., 2021	*Panax quinquefolius*	S	terpenoids	B	6	TC	1.78776	2.00816	0.04897	0.09796
81	Ran et al., 2021	*Panax quinquefolius*	S	terpenoids	B	6	TC	1.78776	1.73878	0.04897	0.04898
21	Xie et al., 2018	*Glycyrrhiza uralensis*	S	terpenoids	B	5	WUE	2.08777	5.88715	1.682328104	0.252340271
22	Xie et al., 2018	*Glycyrrhiza uralensis*	S	terpenoids	B	5	WUE	3.32915	9.04702	1.724366182	1.640245304
23	Xie et al., 2018	*Glycyrrhiza uralensis*	S	terpenoids	B	5	WUE	1.84326	7.33542	1.219663278	2.649651111
24	Xie et al., 2018	*Glycyrrhiza uralensis*	S	flavonoids	B	5	WUE	2.08777	5.88715	1.682328104	0.252340271
25	Xie et al., 2018	*Glycyrrhiza uralensis*	S	flavonoids	B	5	WUE	3.32915	9.04702	1.724366182	1.640245304
26	Xie et al., 2018	*Glycyrrhiza uralensis*	S	flavonoids	B	5	WUE	1.84326	7.33542	1.219663278	2.649651111
55	Hristozkova et al., 2018	*Ocimum basilicum*	S	flavonoids	A	9	WUE	1.88	3.24	0.188	0.324

Note: S: Single AMF inoculation; M: Multi-AMF inoculation; A: Aboveground organs; B: Belowground organs.

Table S3 Overall heterogeneity test

Compounds	I^2 test	df	P(Q test)	Physiological variables	I^2 test	df	P(Q test)
Total	98.73	232	<0.01	Total	99.89	112	<0.01
Aboveground	98.69	100	<0.01	Aboveground	99.91	34	<0.01
Belowground	98.6	131	<0.01	Belowground	99.81	77	<0.01

Table S4 Heterogeneity test on compounds and physiological variables

Compounds	I^2 test	df	P(Q test)	Physiological variables	I^2 test	df	P(Q test)
terpenoids	99.46	40	<0.01	Chl-A	99.87	18	<0.01
flavonoids	98.3	26	<0.01	Chl-B	99.88	18	<0.01
others	98.81	14	<0.01	CHO	0	8	0.8262
phenols	95.79	73	<0.01	Gs	98.99	20	<0.01
alkaloids	98.81	17	<0.01	Pn	98.43	20	<0.01
quinones	69.34	47	<0.01	TC	95.75	16	<0.01
organic acids	64.18	7	<0.01	WUE	55.64	6	<0.01
